# Aerosol Box Use in Reducing Health Care Worker Contamination During Airway Procedures (AIRWAY Study): A Simulation-Based Randomized Clinical Trial

**DOI:** 10.1001/jamanetworkopen.2023.7894

**Published:** 2023-04-12

**Authors:** Adam Cheng, Jonathan Pirie, Yiqun Lin, Carl Y. Lo, Jennifer Davidson, Todd Chang, Clyde Matava, Michael Buyck, Dana Singer Harel, Natasha Collia, Guylaine Neveu, Stephanie Pellerin, Mohsen Madadi, Keya Manshadi, Brandi Wan, Arielle Levy

**Affiliations:** 1 Departments of Pediatrics and Emergency Medicine, Cumming School of Medicine, University of Calgary, Calgary, Alberta, Canada; 2 Department of Pediatrics, The Hospital for Sick Children, University of Toronto, Toronto, Ontario, Canada; 3 KidSIM-ASPIRE Simulation Research Program, Alberta Children’s Hospital, University of Calgary, Calgary, Alberta, Canada; 4 Department of Anesthesiology and Critical Care Medicine, Children’s Hospital of Los Angeles, University of Southern California, Los Angeles; 5 Department of Emergency Medicine, Children’s Hospital of Los Angeles, University of Southern California, Los Angeles; 6 Department of Anesthesiology, The Hospital for Sick Children, University of Toronto, Toronto, Ontario, Canada; 7 Department of Mother and Child Medicine, Division of Pediatric Emergency Medicine, Geneva University Hospitals and University of Geneva, Switzerland; 8 Department of Respiratory Therapy, Sainte-Justine Hospital Mother and Child Simulation Center, CHU Sainte-Justine, Université de Montréal, Montréal, Quebec, Canada; 9 Department of Emergency Medicine, Children’s Hospital of Los Angeles, Los Angeles, California

## Abstract

**Importance:**

The aerosol box has been used during the management of patients with COVID-19 to reduce health care practitioner (HCP) exposure during aerosol-generating medical procedures (AGMPs). Little is known about the effect of aerosol box use on HCP contamination and AGMP procedure time.

**Objective:**

To investigate whether use of an aerosol box during AGMPs reduces HCP contamination or influences the time to successful completion and first-pass success rate for endotracheal intubation (ETI) and laryngeal mask airway (LMA) insertion.

**Design, Setting, and Participants:**

This multicenter, simulation-based, randomized clinical trial was conducted from May to December 2021 at tertiary care pediatric hospitals. Participant teams performed 3 simulated patient scenarios: bag-valve-mask ventilation, ETI, and LMA insertion. During the scenarios, aerosols were generated using Glo Germ. Teams of 2 HCPs were randomly assigned to control (no aerosol box) or intervention groups (aerosol box). Statistical analysis was performed from July 2022 to February 2023.

**Interventions:**

The aerosol box (or SplashGuard CG) is a transparent, plastic barrier covering the patient’s head and shoulders with access ports allowing HCPs to manage the airway.

**Main Outcomes and Measures:**

The primary outcome was surface area of contamination (AOC) on participants. Secondary outcomes were time to successful completion and first-pass success rates for ETI and LMA insertion.

**Results:**

A total of 64 teams (128 participants) were enrolled, with data from 61 teams (122 participants) analyzed. Among the 122 participants analyzed, 79 (64.8%) were female and 85 (69.7%) were physicians. Use of an aerosol box was associated with a 77.5% overall decreased AOC to the torso (95% CI, −86.3% to −62.9%; *P* < .001) and a 60.7% overall decreased AOC to the facial area (95% CI, −75.2% to −37.8%; *P* < .001) in airway HCPs. There was no statistically significant difference in surface contamination after doffing personal protective equipment between groups. Time to completing ETI was longer in the aerosol box group compared with the control group (mean difference: 10.2 seconds; 95% CI, 0.2 to 20.2 seconds; *P* = .04), but there was no difference between groups for LMA insertion (mean difference: 2.4 seconds; 95% CI, −8.7 to 13.5 seconds; *P* = .67).

**Conclusions and Relevance:**

In this randomized clinical trial of aerosol box use in AGMPs, use of an aerosol box reduced contamination deposition on HCPs’ torso and face predoffing; the use of an aerosol box delayed time to successful intubation. These results suggest that the incremental benefits of reduced surface contamination from aerosol box use should be weighed against delayed time to complete intubation, which may negatively affect patient outcome.

**Trial Registration:**

ClinicalTrials.gov Identifier: NCT04880668

## Key Points

**Question:**

Does the use of an aerosol box during airway procedures reduce health care practitioner contamination or influence time to completion of airway tasks?

**Findings:**

In this randomized clinical trial of 61 teams (122 participants) performing aerosol-generating medical procedures, health care practitioners using an aerosol box had significantly less contamination deposited to the torso (predoffing) across all 3 airway procedures compared with those without an aerosol box. There was no significant difference between groups in surface contamination after doffing personal protective equipment.

**Meaning:**

These results suggest that aerosol box use may be protective against surface contamination prior to doffing, but this effect is not maintained after doffing personal protective equipment.

## Teaser

This randomized clinical trial examines whether an aerosol box during aerosol-generating medical procedures reduces health care practitioner contamination or influences the time to successful completion and first-pass success rate for endotracheal intubation and laryngeal mask airway insertion.

## Introduction

The COVID-19 pandemic has presented a threat to the health of health care practitioners (HCPs).^1^ Aerosol-generating medical procedures (AGMPs), such as bag-valve-mask (BVM) ventilation, laryngeal mask airway (LMA) insertion, and endotracheal intubation (ETI) are commonly required for patients with COVID-19 patients who are critically ill.^2,3^ AGMPs produce airborne particles, contributing to the heightened risk of infection among HCPs.^4,5^ Aerosol box devices were developed to shield HCPs from aerosols and potentially minimize exposure to viral particles.^6,7,8,9,10,11^

The aerosol box has been implemented by some hospitals worldwide for management of COVID-19 patients who are critically ill.^12,13,14,15,16,17,18,19^ Studies demonstrate that aerosol box use potentially reduces spread of aerosolized particles,^20,21^ but is often associated with technical challenges.^22,23,24,25^ Simulation-based studies describe delayed time to intubation with aerosol box use, but clinical studies largely demonstrate no difference in intubation times.^7^ Studies have focused primarily on the task of ETI, with little evidence describing aerosol box use during LMA insertion or BVM ventilation.^7,8,9,10,11^ Many existing studies were underpowered, provided minimal aerosol box training, or recruited participants as solitary airway practitioners, making it difficult to determine the true value of aerosol boxes.

This study evaluated the effectiveness of aerosol box use for reducing HCP contamination during performance of AGMPs (BVM ventilation, LMA insertion, and ETI) by a trained airway team during care of simulated patients in respiratory failure. We also determined if aerosol box use influenced the time to successful completion and first-pass success rate for ETI and LMA insertion, and if other factors, such as HCP sex, height, and clinical experience influenced practitioner contamination or time to AGMP completion.

## Methods

We conducted a prospective, multicenter, simulation-based randomized clinical trial. Research ethics board approval was secured from all study sites, and informed consent was obtained by collecting written consent from all participants. This report follows the Consolidated Standards of Reporting Trials (CONSORT) reporting guideline for simulation-based randomized clinical trials.^26^

### Study Participants

Participants were recruited from intensive care units, operating rooms, and emergency departments of 4 tertiary care pediatric hospitals to participate in 3 airway scenarios. Participants were consented and recruited in teams of 2 for the roles of airway practitioner and airway assistant. Inclusion criteria for the airway practitioners were: (1) Attending physician in emergency medicine, intensive care, pediatrics, or anesthesia; and (2) Adult or Pediatric Advanced Life Support certification. Inclusion criteria for airway assistants were the same as the aforementioned, but also included residents, fellows, nurses, and respiratory therapists. Participants were excluded if they were unable to perform the tasks required of the role assigned.

### Intervention

#### Aerosol Box

The aerosol box (or SplashGuard CG) is a transparent, plastic barrier covering the patient’s head and shoulders, with 6 circular access ports on the front and sides allowing practitioners access to manage the airway^17,27^ (eFigure 1 in Supplement 1). A plastic drape extending from the top of the box down to the patient’s chest prevents spread of aerosols.^15,17,28^ Suction tubing at the head of the aerosol box was added, as prior studies have demonstrated that continuous suction decreases airborne particle exposure.^20,28,29,30,31^ Wall suction was set at 200 mm Hg, measured to generate a negative airflow of approximately 50 L/min.

### Study Procedures

**Figure 1.**
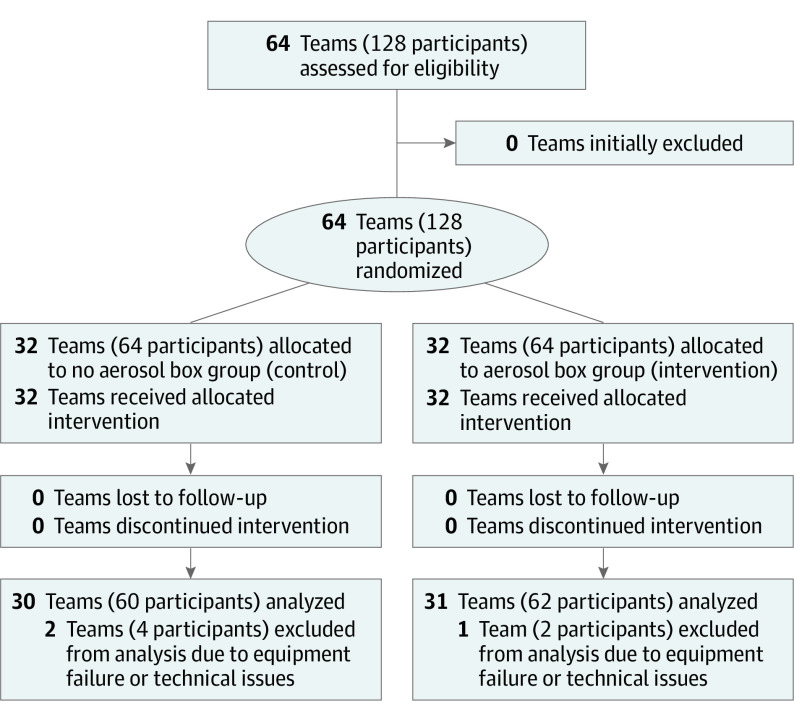
Consolidating Standards of Reporting Trials (CONSORT) Diagram^a^ ^a^Three teams excluded due to equipment failure or technical issues.

Participants were randomized by teams of 2 using an online randomizer tool into either the control group or intervention group. Airway practitioners were paired with airway assistants in a nonrandom fashion. Randomization occurred at the level of the team, was stratified by study site, and conducted in blocks of 4 to ensure equal distribution of teams across study groups. Study packages were prepared with opaque envelopes and administered by site research coordinators to achieve allocation concealment. See Figure 1 for the CONSORT flow diagram.

#### Aerosol Box Training

All intervention group participants viewed a 10-minute aerosol box orientation video demonstrating BVM ventilation, ETI, and LMA insertion with a 2-person airway team using an aerosol box. Participant pairs then practiced all 3 AGMPs using a rapid cycle deliberate practice model,^17,32^ paired with repeated opportunity to practice coupled with checklist-guided feedback (eTables 1-3 in Supplement 1) from an airway expert (maximum 15 minutes). Control group participants did not view the video but were given 15 minutes to practice each airway procedure without the use of an aerosol box.

#### Simulating Aerosolization

This study used an aerosolization model first described by Oman et al,^33^ capable of generating measurable particles (0.3 to 10 micrometers) and allowing for performance of AGMPs (eFigure 1 in Supplement 1). To visualize particles, we used Glo Germ (Glo Germ Company, Moab, Utah), a nontoxic, melamine copolymer resin marker that illuminates when exposed to UV-A light.^4,34^ With a particle size of approximately 1 to 5 micrometers, Glo Germ has been used as a surrogate for infectious droplet nuclei.^4,33,34,35,36,37,38^ Glo Germ powder was added to the nebulizer chamber and filled to the top before each scenario; powder was aerosolized when airflow to the nebulizer chamber was turned on. Full details are found in the study protocol (Supplement 2).

#### Simulation Scenarios

After training, all teams participated in 3 sequential simulation scenarios. The order of scenario delivery was randomized to eliminate scenario order as a potential confounder. Prior to each scenario, all participants donned personal protective equipment (PPE), consisting of a gown, nitrile gloves, face shield, goggles, and a N95 respirator. Donning of PPE was guided by a standardized PPE donning checklist and checked by a research assistant prior to all scenarios. The equipment within the resuscitation room and PPE was standardized across all sites. All scenarios were 5 minutes in duration and tightly standardized by using a scenario template with scripted patient progression. The 3 scenarios were: (1) scenario A: BVM ventilation; participants were directed to initiate and continue BVM ventilation for an adolescent patient with progressive respiratory distress from suspected COVID-19; aerosolization of particles occurred prior to and during BVM ventilation (eTable 4 in Supplement 1); (2) scenario B: ETI; same patient as aforementioned with respiratory failure; participants were advised to sedate, paralyze, and intubate the patient using a videolaryngoscope (with a Macintosh blade^39^), and provide manual ventilation after intubation; aerosolization of particles occurred up until a muscle relaxant was administered (eTable 5 in Supplement 1); (3) scenario C: LMA insertion; same patient as in scenario B; participants were directed to sedate, paralyze, and insert an LMA, followed by manual ventilation after LMA insertion; aerosolization was simulated in the same manner as scenario B (eTable 6 in Supplement 1).

Data collection occurred immediately after each scenario, prior to and after doffing of PPE. Doffing was guided by a PPE-doffing checklist and closely monitored by a research facilitator to ensure no cross contamination. A standardized cleaning protocol was used across sites, with the resuscitation room and manikin cleaned between each scenario and new equipment and PPE used for each scenario. All scenarios were videotaped from a birds-eye view angle at the head of the bed.

### Outcome Measures

The primary outcome was the surface area of contamination (AOC), quantified from digital photos of Glo Germ deposited on participants. A digital reference grid was applied over each photo, with mean gray values of areas (illuminated by Glo Germ) within each grid pixel calculated using Image J software.^40^ Digital images of participants were captured in a standardized fashion under UV illumination. Images were taken pre- and postdoffing to capture the extent of contamination on PPE (pre) and on practitioner surfaces underneath PPE (post). Images were analyzed using established methods with supportive validity evidence^41^ (eFigure 2 in Supplement 1). One research team member, blinded to the participant allocation, measured the AOC in the upper extremities, torso, and facial area. Predoffing AOCs were presented as millimeters squared, and the postdoffing AOCs were presented dichotomously as clean (ie, no contamination) or contaminated (ie, ≥1 mm^2^ contamination). Secondary outcomes were: (1) time to successful intubation, measured as time from removal of face mask to first effective ventilation after passing the endotracheal tube; (2) time to successful LMA insertion, measured as time from removal of face mask to time to first effective ventilation; and (3) first-pass success rate for ETI and LMA insertion. Secondary outcomes were extracted via video review by 3 trained video reviewers with expertise in airway management. Blinding video reviewers was not possible due to the nature of the intervention.

### Statistical Analysis

Sample size estimation was based on the primary outcome measure. As each team received repeated measures (ie, 3 scenarios), to achieve a significance level of 0.05, a power of 0.8, and a high intracluster correlation coefficient (ρ = 0.7), a sample size of 60 teams (120 participants) in total, or 30 teams per study group, was required to detect a medium effect size (Cohen *d* = 0.65). Accounting for missing data due to technical issues, we recruited 2 extra teams per study group, resulting in a total sample size of 64 teams (128 participants).

Demographic characteristics of participants were summarized with descriptive statistics. Analyses were conducted using R software and lme4 packages^42^ with 2-sided *P* < .05 considered statistically significant. For the primary outcome, due to the nonnormal distribution of data, log-transformations were conducted, and mixed-effect linear regression models were used to evaluate the overall effect of aerosol box use on predoffing AOC (fixed effect: aerosol box, random intercept) for both airway practitioners and assistants. Linear regression models were used to examine the procedure-specific effect of intervention. In addition to model coefficients, we presented the geometric means of the estimates and their 95% CIs by exponentiating the values estimated in the model.^43^ Fisher exact tests were used to compare the proportion of clean participants between the groups. Time to procedural completion and first-pass success rates were compared with 2-sample *t* tests and Fisher exact tests, respectively. Secondary analyses were conducted to explore factors other than aerosol box use that could influence procedure time, using mixed-effect linear regression models (fixed effect: gender, experience, body metrics, and aerosol box; random intercept). Statistical analysis was performed using R software version 4.2.2 (R Project for Statistical Computing) from July 2022 to February 2023.

## Results

### Demographics

Sixty-four teams (128 participants) were recruited between May 2021 and December 2021. Data from 3 teams (ie, 2 in control group, 1 in intervention group) were excluded due to technical issues (ie, failure to save the images or record the videos). Data from the remaining 61 teams (n = 30 control, n = 31 intervention) were included in the analysis. Among 122 participants, 79 (64.8%) were female, 42 (34.4%) were male, and 1 (0.8%) preferred not to answer; 85 (69.7%) were physicians, 15 (12.3%) were nurses, and 22 (18.0%) were respiratory therapists (Table 1).

**Table 1.** Demographic Characteristics

Characteristic	Control, No. (%) (n = 60)	Intervention, No. (%) (n = 62)
Gender		
Male	22 (36.7)	20 (32.3)
Female	38 (63.3)	41 (66.1)
Prefer not to answer	0	1 (1.6)
Profession		
Physician	40 (66.7)	45 (72.6)
Nurse	11 (18.3)	4 (6.5)
Respiratory therapist	9 (15.0)	13 (21.0)
Area of practice		
Pediatric acute care^a^	47 (78.4)	51 (82.2)
Adult acute care^a^	8 (13.3)	6 (9.7)
General Pediatrics (nonacute care)	5 (8.3)	5 (8.1)
Body metrics, mean (SD)		
Height, cm	169.4 (10.0)	170.5 (10.5)
Weight, kg	69.5 (12.5)	71.3 (17.2)
Body surface area, m^2^	1.79 (0.19)	1.82 (0.24)
**Experience in the past 12 mo**		
Direct laryngoscopy intubation		
Yes	41 (68.3)	41 (66.1)
No	19 (31.7)	21 (33.9)
Video laryngoscopy intubation		
Yes	36 (60.0)	35 (56.5)
No	24 (40.0)	27 (43.5)
Laryngeal mask airway insertion		
Yes	28 (46.7)	23 (37.1)
No	32 (53.3)	39 (62.9)
Bag-valve-mask ventilation		
Yes	49 (81.7)	43 (69.4)
No	11 (18.3)	19 (30.6)
Multiple medical procedures (>5) in a neonatal isolette		
Yes	8 (13.3)	9 (14.5)
No	52 (86.7)	53 (85.5)
Knowledge and experience about aerosol box		
Never heard about it	10 (16.7)	5 (8.1)
Have read about it	24 (40.0)	31 (50.0)
Have seen how it works	10 (16.7)	10 (16.1)
Have used it in simulated events	13 (21.7)	12 (19.4)
Have used it in real events	3 (5.0)	4 (6.5)

^a^ Acute care includes emergency medicine, critical care, and anesthesiology.

### Practitioner Contamination: Predoffing

Compared with the control group, use of the aerosol box was significantly associated with a 65.0% overall increased AOC in airway practitioners (geometric mean AOC: 680.7 vs 1124.3 millimeters squared; 65.0% increase; 95% CI, 7.1%-154.6%; *P* = .02) and a 142.4% increased AOC in airway assistants (geometric mean AOC: 121.6 vs 296.4 millimeters squared; 142.4% increase; 95 % CI, 34.0%-339.2%; *P* = .004) in the upper extremities. All procedure-specific effects of aerosol box use on AOC in the upper extremities were not statistically significant (Table 2; eTable 7, eTable 8, eFigure 3 in Supplement 1).

**Table 2.** Predoffing AOC

	AOC, median (IQR), mm^2^		Geometric mean AOC (95% CI), mm^2^^a^		Difference, % (95% CI)^b^	*P* value
	Control	Intervention	Control	Intervention		
**Airway practitioner**						
Overall effect						
Upper extremity	1444.1 (528.3-4465.7)	4169.9 (595.7-8797.2)	680.7 (78.3 to 5817.8)	1124.3 (129.8 to 9596.5)	65.0 (7.1 to 154.6)	.02
Torso	5.9 (0.5–94.0)	0.5 (0.1-5.3)	9.9 (2.3 to 34.7)	1.4 (−0.3 to 7.0)	−77.5 (−86.3 to −62.9)	<.001
Face	0	0	1.7 (0.1 to 5.4)	0.1 (−0.6 to 1.5)	−60.7 (−75.2 to −37.8)	<.001
BVM						
Upper extremity	751.0 (248.7-4302.8)	3348.0 (347.5-5760.3)	498.8 (187.7 to 1322.6)	1026.1 (393.0 to 2676.4)	105.5 (−47.5 to 705.6)	.30
Torso	6.1 (0.7-36.5)	0.6 (0.1-11.1)	8.9 (4.1 to 18.4)	2.5 (0.1 to 6.2)	−64.6 (−86.2 to −9.6)	.03
Face	0.0 (0.0-6.1)	0	2.6 (0.8 to 6.2)	0.4 (0.0 to 5.6)	−60.2 (−85.0 to 5.1)	.06
ETI						
Upper extremity	1465.1 (573.0-5491.9)	7275.6 (481.2-11 384.0)	1481.1 (781.7 to 2805.2)	2821.7 (1505.3 to 5288.4)	90.4 (−22.2 to 366.3)	.15
Torso	3.8 (0.2-181.8)	0.4 (0.0-2.4)	13.2 (5.8 to 28.4)	1.4 (1.1 to 15.1)	−82.7 (−93.7 to −52.3)	.001
Face	0	0	1.7 (0.5 to 3.7)	0.1 (0.1 to 4.2)	−57.8 (−80.9 to −6.6)	.03
LMA						
Upper extremity	2038.5 (729.4-4990.7)	6048.7 (1666.5-9149.1)	2044.0 (1049.4 to 3980.5)	2752.8 (1428.8 to 5302.7)	34.6 (−47.1 to 242.8)	.52
Torso	8.9 (0.5-110.3)	0.7 (0.0-8.7)	14.5 (6.6 to 30.9)	2.6 (0.8 to 6.2)	−77.1 (−91.6 to −37.4)	.004
Face	0	0	2.0 (0.6 to 4.5)	0.2 (0.1 to 5.0)	−60.5 (−83.3 to −6.2)	.04
**Airway assistant**						
Overall effect						
Upper extremity	431.4 (53.4-2121.3)	1591.1 (179.8-5606.7)	121.6 (7.5 to 1748.5)	296.4 (19.6 to 4234.4)	142.4 (34.0 to 339.2)	.004
Torso	18.1 (1.0–117.5)	1.9 (0.0-18.5)	11.6 (1.5 to 63.2)	2.7 (−0.3 to 17.6)	−70.9 (−81.6 to −54.1)	<.001
Face	0	0	0.1 (−0.1 to 0.4)	0.1 (−0.1 to 0.3)	−2.8 (−20.1 to 18.4)	.78
BVM						
Upper extremity	81.3 (6.5-311.8)	587.9 (12.0-3503.1)	63.6 (22.0 to 180.6)	217.6 (78.0 to 603.8)	238.6 (−20.6 to 134.4)	.10
Torso	9.9 (0.3-66.6)	1.8 (0.0-28.1)	9.8 (4.4 to 20.9)	4.4 (1.7 to 9.7)	−50.4 (−81.4 to 33.1)	.16
Face	0	0	0.3 (0.0 to 0.9)	0.2 (−0.2 to 0.6)	−11.3 (−45.2 to 43.7)	.62
ETI						
Upper extremity	1073.0 (113.5-4183.6)	3684.1 (199.6-8469.8)	487.0 (187.0 to 1265.3)	1165.1 (455.4 to 2978.3)	138.9 (−37.2 to 810.3)	.20
Torso	25.4 (1.8-105.8)	6.9 (0.1-30.2)	17.3 (8.1 to 35.8)	6.0 (2.5 to 12.8)	−61.9 (−85.7 to 1.3)	.05
Face	0	0	0.1 (−0.2 to 0.4)	0.2 (−0.4 to 0.4)	6.1 (−27.0 to 54.3)	.75
LMA						
Upper extremity	758.8 (129.5-3514.7)	1833.3 (279.8-5506.1)	412.8 (157.5 to 1079.2)	959.6 (372.8 to 2468.0)	132.1 (−39.5 to 791.9)	.22
Torso	24.1 (2.8-164.7)	1.5 (0.0-10.3)	25.4 (11.4 to 54.9)	3.3 (1.1 to 8.0)	−83.6 (−94.3 to −53.1)	.001
Face	0	0	−0.01 (−0.03 to 0.01)	0.00 (−0.01 to 0.04)	−1.2 (−3.6 to 1.2)	.31

Abbreviations: AOC, area of contamination; BVM, bag-valve-mask ventilation; ETI, endotracheal intubation; LMA, laryngeal mask airway.

^a^ Geometric mean of AOC calculated by exponentiating the mean of log-AOC, then subtracting 1. Therefore, the 95% CIs are not symmetric. See eTable 7 and eTable 8 in Supplement 1 for estimation of log-AOC and difference of log-AOC between groups.

^b^ Relative difference calculated by exponentiating the mean difference of log-AOC and subtracting 1, then multiplying by 100%. For example, the relative difference of torso contamination reported in the overall model (−70.9%) is interpreted as, compared with control group, the use of aerosol box is associated with a 70.9% lower geometric mean of AOC.

Compared with control group, use of aerosol box was significantly associated with a 77.5% overall decreased AOC to torso in airway practitioners (geometric mean AOC: 9.9 vs 1.4 mm^2^; 77.5% decrease; 95% CI, −86.3% to −62.9%; *P* < .001) and a 70.9% overall decreased AOC to torso in airway assistant (geometric mean AOC: 11.6 vs 2.7 mm^2^; 70.9% decrease; 95% CI, −81.6% to −54.1%; *P* < .001). All procedure-specific effects of aerosol box use on AOC to the torso remained significant among airway practitioners, but only significant in LMA insertion among airway assistants (Table 2; eTable 7, eTable 8, eFigure 4 in Supplement 1).

Very few airway practitioners and airway assistants had contamination to the facial area across all 3 procedures (median [IQR] AOC = 0 [0-0] mm^2^ across all 3 procedures). Compared with controls, use of an aerosol box was significantly associated with an overall 60.7% decreased AOC to the facial area of airway practitioners (geometric mean AOC: 1.7 vs 0.1 mm^2^; 60.7% decrease; 95% CI, −75.2% to −37.8%; *P* < .001). The overall effect of aerosol box use was not significant among airway assistants (geometric mean AOC: 0.1 vs 0.1 mm^2^; relative mean difference: −2.8%; 95% CI, −20.1% to 18.4%; *P* = .78) (Table 2; eTable 7, eTable 8, eFigure 5 in Supplement 1).

### Practitioner Contamination: Postdoffing

Most participants had no contamination to the body after doffing PPE (BVM: 121 of 122 [99.2%]; ETI: 119 of 122 [97.5%]; LMA: 121 of 122 [99.2%]). There was no statistically significant difference in the percentage of participants with postdoffing contamination between groups (eTable 9 in Supplement 1).

### Time to Procedural Completion and First-Pass Success Rates

**Figure 2.**
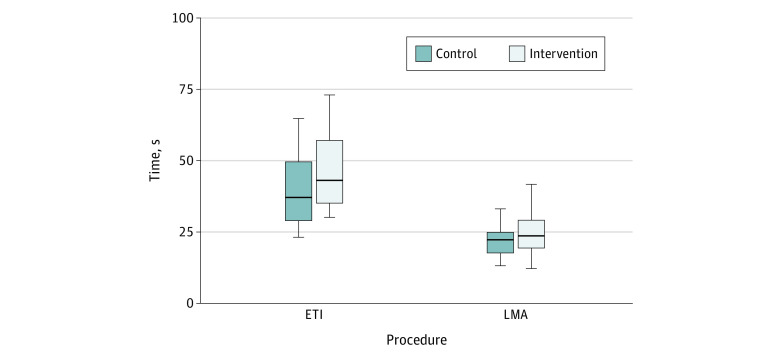
Time to Procedural Completion The bold line in the center indicates the median, the size of the box indicates interquartile ranges, and the whiskers indicate the minimum and maximum. ETI indicates endotracheal intubation; LMA, laryngeal mask airway.

Participants using the aerosol box spent a significantly longer time to complete ETI than control group teams (mean [SD] ETI time for control group: 40.1 [14.2] seconds) vs intervention group: 50.3 [23.5] seconds; mean difference: 10.2 seconds; 95% CI, 0.2-20.2 seconds; *P* = .04). Time to LMA insertion was not significantly different between the control and intervention groups (mean [SD] LMA time in control group: 29.1 [26.5] seconds vs intervention group: 26.7 [15.1] seconds; mean difference: 2.4 seconds; 95% CI, −8.7 to 13.5 seconds; *P* = .67). There was no significant difference between groups for first-pass success rates for ETI (control vs intervention count: 30 of 30 [100%] vs 30 of 31 [96.8%]; rate ratio [RR], 1.03; 95% CI, 0.97 to 1.10; *P* > .99) and LMA insertion (control vs intervention count: 29 of 30 [96.7%] vs 30 of 31 [96.8%]; RR, 1.00; 95% CI, 0.91 to 1.10; *P* > .99) (Figure 2).

### Factors Influencing Time to Airway Insertion

Aerosol box use was found to be independently associated with longer intubation time (adjusted mean difference: 12.6 seconds; 95% CI, 2.0 to 23.2 seconds; *P* = .02). Other factors such as gender, year of experience, and adjusted height (difference between practitioner height and stretcher height) were not significantly associated with intubation time. No factors were found to be associated with the LMA insertion time (Table 3).

**Table 3.** Factors Associated With Procedural Completion Time

Factors	ETI		LMA	
	Adjusted coefficient (95% CI)	*P* value	Adjusted coefficient (95% CI)	*P* value
Intercept	44.5 (6.9 to 82.0)	.02	3.5 (−39.8 to 46.8)	.87
Group				
Control	[Reference]	NA	[Reference]	NA
Intervention	12.6 (2.0 to 23.2)	.02	−3.3 (−15.5 to 9.0)	.60
Gender				
Female	[Reference]	NA	[Reference]	NA
Male	4.7 (−3.3 to 12.6)	.24	−0.5 (−9.5 to 8.4)	.91
Experience (year)	0.4 (−0.2 to 1.0)	.22	−0.2 (−0.9 to 0.5)	.63
Adjusted height, cm	−0.2 (−0.6 to 0.3)	.51	0.3 (−0.2 to 0.9)	.20

Abbreviations: ETI, endotracheal intubation; LMA, laryngeal mask airway insertion.

## Discussion

This study quantified practitioner contamination after performance of BVM, LMA insertion, and ETI, finding that aerosol box use reduces surface contamination significantly on the torso and face of airway practitioners predoffing, with little difference in the contamination of upper extremities (predoffing) in the intervention group. We found minimal amounts of surface contamination postdoffing in both control and intervention groups. Existing literature has described technical challenges associated with box use resulting in prolonged intubation times,^7,21,23,25,37,44,45,46,47,48^ raising concerns about patient safety particularly with untrained airway practitioners.^31^ This study sought to optimize practitioner performance by training airway teams to use the aerosol box. We demonstrated that aerosol box use prolonged the time to intubation but did not affect time to LMA insertion. These results help clarify the true utility of aerosol boxes during AGMPs, bringing into question whether routine use is justifiable.

The aerosol box was designed to mitigate HCP infection by providing a physical barrier to minimize exposure to aerosols.^7,8,9,11,17,18^ This study showed that aerosol box use reduces surface contamination on the PPE (torso and face) of airway practitioners after performing AGMPs. In a prior manikin-based study (with continuous aerosolization), Noor Azhar et al^21^ examined aerosol box during videolaryngoscopic intubation among emergency medicine trainees and found that use of an aerosol box resulted in fewer contaminated body regions predoffing but no difference in the number of contaminated regions postdoffing. In this study, aerosols were produced with each patient respiration, allowing for greater spread of Glo Germ with positive pressures. The aerosol box was effective at containing aerosols within the box, resulting in greater surface contamination on the upper extremities (predoffing). In both groups, there was virtually no surface contamination postdoffing. Even though existing evidence supports the protective value of aerosol boxes for HCPs,^21,28,49^ the provision of PPE with effective doffing appears to offset this value. Perhaps the main benefit aerosol box use comes with reduced PPE contamination to the facial region of airway practitioners; but one could argue this finding to be less important in the context of immunized practitioners using a face shield and an N95 mask.

In this study, teams of trained airway practitioners using an aerosol box showed increased time to intubation, but no difference in time to LMA insertion or first-pass success rates (for ETI and LMA insertion) compared with teams without an aerosol box. While prior manikin-based studies report slightly longer time to intubation with aerosol box use,^7,21,23,25,37,44,45,46,47,48^ certain study design elements such as a single airway practitioner^23,44,47^ or a lack of prior aerosol box training^23,45,46^ may have contributed to delayed intubation times. Data from studies assessing intubation in real patients is mixed. Two prospective trials showed no difference in time to intubation^50,51^ or first-pass success rate^50^ with aerosol box use, but a survey of practitioners who had used an aerosol box during intubation reported only a 78.9% first-pass success rate.^18^ Aerosol box use during intubation requires an increased number of optimization procedures and hand movements, along with hindering visibility for some practitioners.^22,23,24,25^ In theory, practicing in pairs should provide sufficient opportunity to overcome these challenges. However, even our group of trained airway teams had prolonged intubation times, which may translate to suboptimal outcomes in real patients with severe physiological derangements. Ultimately, the true value of the aerosol box use as a protective measure should consider multiple variables, including local incidence of infection, severity of infection, practitioner immunization rate, and provision of PPE.

### Limitations

This study has several limitations. The amount of aerosolization that occurs in patients in clinical practice is highly variable and dependent upon many factors, making it impossible to exactly replicate aerosolization. Creating a model that was standardized (ie, particle type, airflow, ventilation pressure) and capable of generating substantial contamination allowed comparisons across a variety of different clinical contexts. The scenarios were limited to the performance of the airway procedure, so study results do not consider other aspects of patient care required for patients who are critically ill. Also, blinding video reviewers to the intervention was not possible, so this may have introduced potential bias, which was mitigated by conducting reviewer training and blinding to participant identity.

## Conclusions

The results of this randomized clinical trial suggest that aerosol box use reduces surface contamination on the torso and face of airway practitioners (predoffing) and prolongs time to intubation, but makes no difference in practitioner contamination postdoffing. Future work should explore the effect of aerosol box use in simulated and real patient care, with outcomes including process of care measures, patient physiology, patient outcomes, and practitioner infection rates.

## Notes

Supplement 1.
eMethods.

eResults.
**eFigure 1.** Aerosol Box Setup for Airway Management Simulation **eFigure 2.** Sample Images of Provider Contamination **eFigure 3.** Median Area of Contamination to Upper Extremity (Airway Providers and Assistants) **eFigure 4.** Median Area of Contamination to Torso (Airway Providers and Assistants) **eFigure 5.** Proportion of Participants With Contamination to Facial Area (Airway Providers and Assistants) **eTable 1.** Bag Valve Mask Training Checklist **eTable 2.** Endotracheal Intubation Training Checklist **eTable 3.** Laryngeal Mask Airway Training Checklist **eTable 4.** Scenario – Bag Valve Mask Ventilation **eTable 5.** Scenario – Endotracheal Intubation **eTable 6.** Scenario – Laryngeal Mask Airway Insertion **eTable 7.** Pre-Doffing Area of Contamination of Airway Providers (Model Coefficient) **eTable 8.** Pre-Doffing Area of Contamination of Airway Assistants (Model Coefficient) **eTable 9.** Proportion of Participants With Contamination (Post-Doffing) **eTable 10.** Factors Associated With Area of Contamination to Healthcare Workers (Pre-Doffing)

Supplement 2.
Trial Protocol

Supplement 3.
Nonauthor Collaborators

Supplement 4.
Data Sharing Statement
