# Supplementary material for: Aerosol Box Use in Reducing Health Care Worker Contamination During Airway Procedures (AIRWAY Study): A Simulation-Based Randomized Clinical Trial
Source: JAMA Netw Open. 2023 Apr 12;6(4):e237894. doi: 10.1001/jamanetworkopen.2023.7894 (PMC10099073; doi:10.1001/jamanetworkopen.2023.7894)
Supplement: Supplement 1. — eMethods. eResults. eFigure 1. Aerosol Box Setup for Airway Management Simulation eFigure 2. Sample Images of Provider Contamination eFigure 3. Median Area of Contamination to Upper Extremity (Airway Providers and Assistants) eFigure 4. Median Area of Contamination to Torso (Airway Providers and Assistants) eFigure 5. Proportion of Participants With Contamination to Facial Area (Airway Providers and Assistants) eTable 1. Bag Valve Mask Training Checklist eTable 2. Endotracheal Intubation Training Checklist eTable 3. Laryngeal Mask Airway Training Checklist eTable 4. Scenario – Bag Valve Mask Ventilation eTable 5. Scenario – Endotracheal Intubation eTable 6. Scenario – Laryngeal Mask Airway Insertion eTable 7. Pre-Doffing Area of Contamination of Airway Providers (Model Coefficient) eTable 8. Pre-Doffing Area of Contamination of Airway Assistants (Model Coefficient) eTable 9. Proportion of Participants With Contamination (Post-Doffing) eTable 10. Factors Associated With Area of Contamination to Healthcare Workers (Pre-Doffing) [file jamanetwopen-e237894-s001.pdf]

Supplemental Online Content

Cheng A, Pirie J, Lin Y, et al; International Network for Simulation-based Pediatric Innovation, Research and Education (INSPIRE) Aerosol-Generating Medical Procedure (AGMP) Investigators. Aerosol box use in reducing health care worker contamination during airway procedures (AIRWAY Study): a simulation-based randomized clinical trial. *JAMA Netw Open.* 2023;6(4):e237894. doi:10.1001/jamanetworkopen.2023.7894

eMethods.

eResults.

eFigure 1. Aerosol Box Setup for Airway Management Simulation

eFigure 2. Sample Images of Provider Contamination

eFigure 3. Median Area of Contamination to Upper Extremity (Airway Providers and Assistants)

eFigure 4. Median Area of Contamination to Torso (Airway Providers and Assistants)

eFigure 5. Proportion of Participants With Contamination to Facial Area (Airway Providers and Assistants)

eTable 1. Bag Valve Mask Training Checklist

eTable 2. Endotracheal Intubation Training Checklist

eTable 3. Laryngeal Mask Airway Training Checklist

eTable 4. Scenario – Bag Valve Mask Ventilation

eTable 5. Scenario – Endotracheal Intubation

eTable 6. Scenario – Laryngeal Mask Airway Insertion

eTable 7. Pre-Doffing Area of Contamination of Airway Providers (Model Coefficient)

eTable 8. Pre-Doffing Area of Contamination of Airway Assistants (Model Coefficient)

eTable 9. Proportion of Participants With Contamination (Post-Doffing)

eTable 10. Factors Associated With Area of Contamination to Healthcare Workers (Pre-Doffing)

This supplemental material has been provided by the authors to give readers additional information about their work.

© 2023 Cheng A et al. *JAMA Network Open.*

eMethods

Study Procedures

In our model, a Resusci Anne™ QCPR manikin (Laerdal Medical, Stavanger, Norway) was combined with a nebulizer (filled with GloGerm™) and Ambu™ bag-valve mask resuscitator (Ambu, Maryland, USA). Within the manikin, the trachea was disconnected from the lung, and securely connected to tubing leading to the Ambu™ bag, with a PEEP valve used to cover the exhaust valve. Additional tubing attached to a nebulizer chamber was connected to an oxygen source, with flow standardized to 8L/min during patient respirations. The self-inflating bag was squeezed by a research facilitator to mimic patient respirations, with titration to peak inspiratory pressures of approximately 20cm H_20. In all scenarios, airflow was turned off and patient respirations ceased after administration of a muscle relaxant. Pilot work done by our research team using this model resulted in contamination on the hands, torso, and face shield of airway providers during BVM ventilation (without an aerosol box), suggesting these methods are sufficient to produce a measurable amount of GloGerm™ particles.

Prior to initiating recruitment for the study, one mock recruitment session (with volunteer participants) from each site was conducted and reviewed to ensure compliance with standardized research protocols.

Reviewer Training

Video reviewer training was conducted using pilot videos, with ICC>0.99 at the end of training. Blinding reviewers to group allocation was not possible (ie. aerosol box visible). Eight videos were randomly selected for triplicate review, demonstrating high inter-rater reliability (ICC=0.99). The remainder of the videos were then randomly assigned to a single reviewer.

eResults

Factors influencing provider contamination

The AOC to the upper extremity area of the participants significantly increased when using the aerosol box (adjusted difference: 111%, 95%CI: 1.4 to 399%, p = 0.05), adjusting for other variables. The AOC to the upper extremities was significantly higher when performing video laryngoscopy intubation (adjusted difference 325%, 95%CI: 190 to 521%, p<0.001) and LMA insertion (adjusted difference: 317%, 95%CI: 185 to 510%, p<0.001), compared to performing BVM only (eTable 10). The AOC to the torso and facial area significantly decreased when using the aerosol box (adjusted difference: -75%, 95%CI: -86 to -55%), adjusting for other confounding factors. Other factors (i.e. provider role, procedure type, BSA) were not significantly associated with the AOC to the torso and facial area. (eTable 10)

© 2023 Cheng A et al. *JAMA Network Open.*

eFigure 1 – Aerosol box setup for airway management simulations

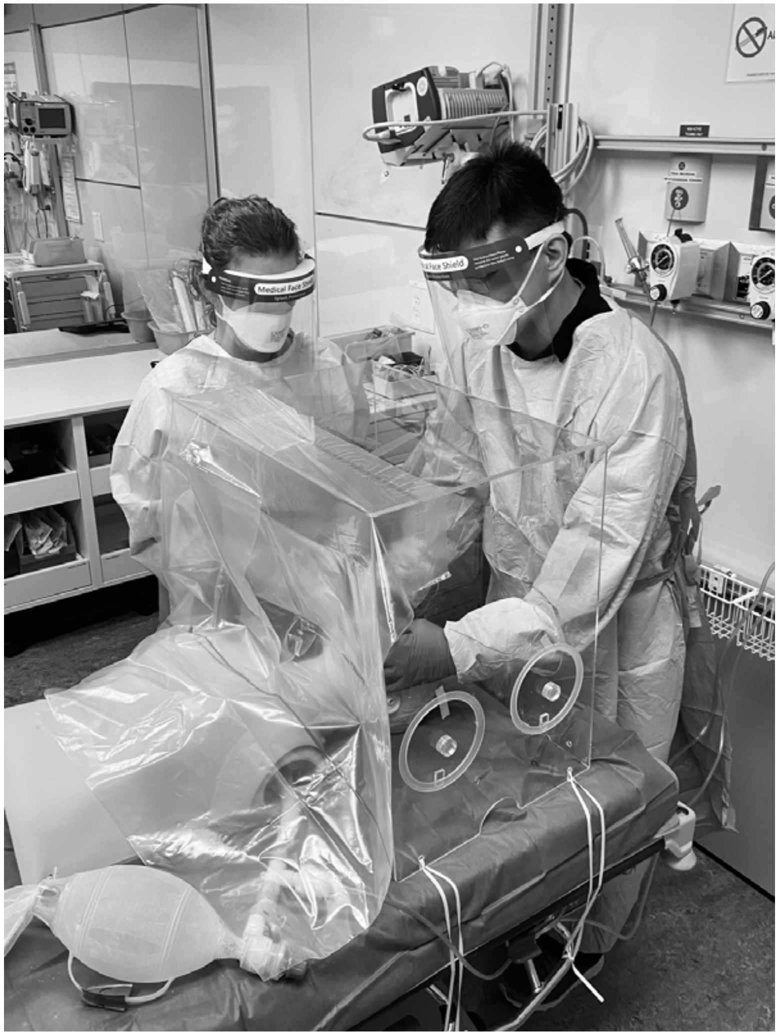

© 2023 Cheng A et al. *JAMA Network Open.*

eFigure 2: Sample images of provider contamination

(a) Photo of an airway provider after BVM procedure in control group (no aerosol box)

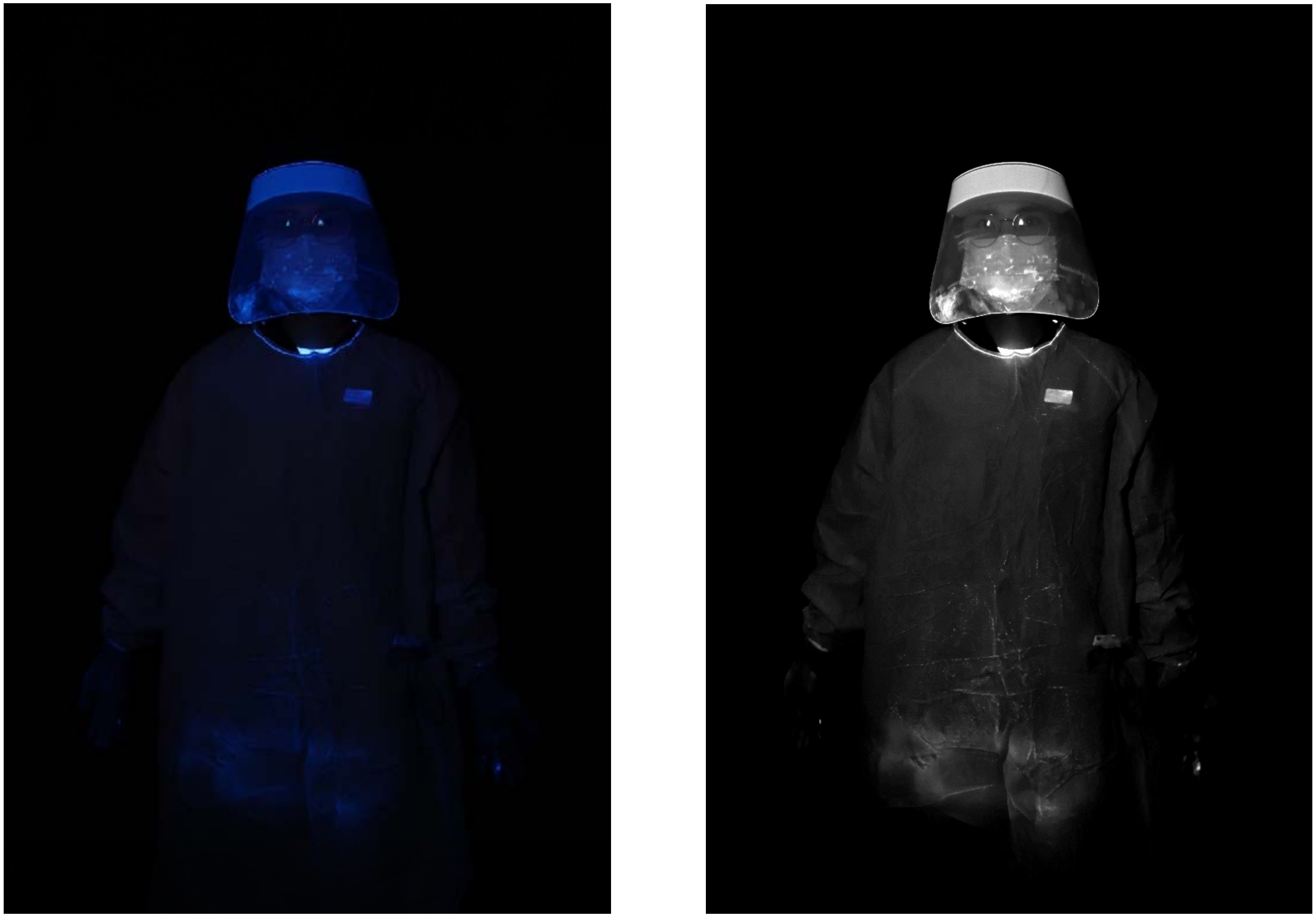

Left: the orignial pictures; Right: brightness and contrast adjusted to better visualize the contamination to hands, torso and face shield

© 2023 Cheng A et al. *JAMA Network Open.*

(b) Photo of an airway provider after the BVM procedure in the intervention group (with aerosol box)

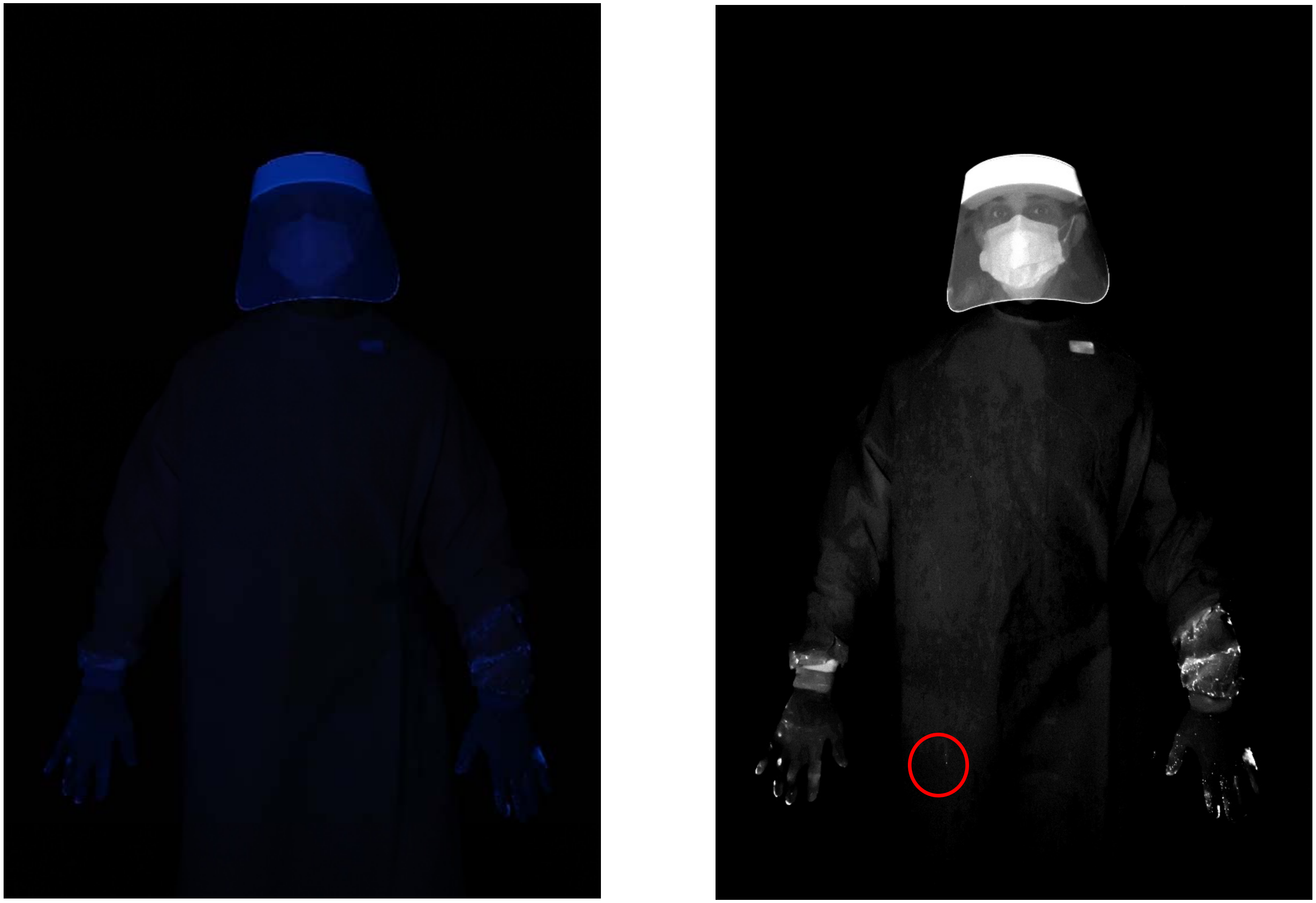

Left: original picture; Right: Brightness and contrast adjusted to better visualize the most contamination to the upper extremities and minimal contamination to the torso (red circle)

© 2023 Cheng A et al. *JAMA Network Open.*

eFigure 3: Median Area of contamination to upper extremity (airway providers and assistants)

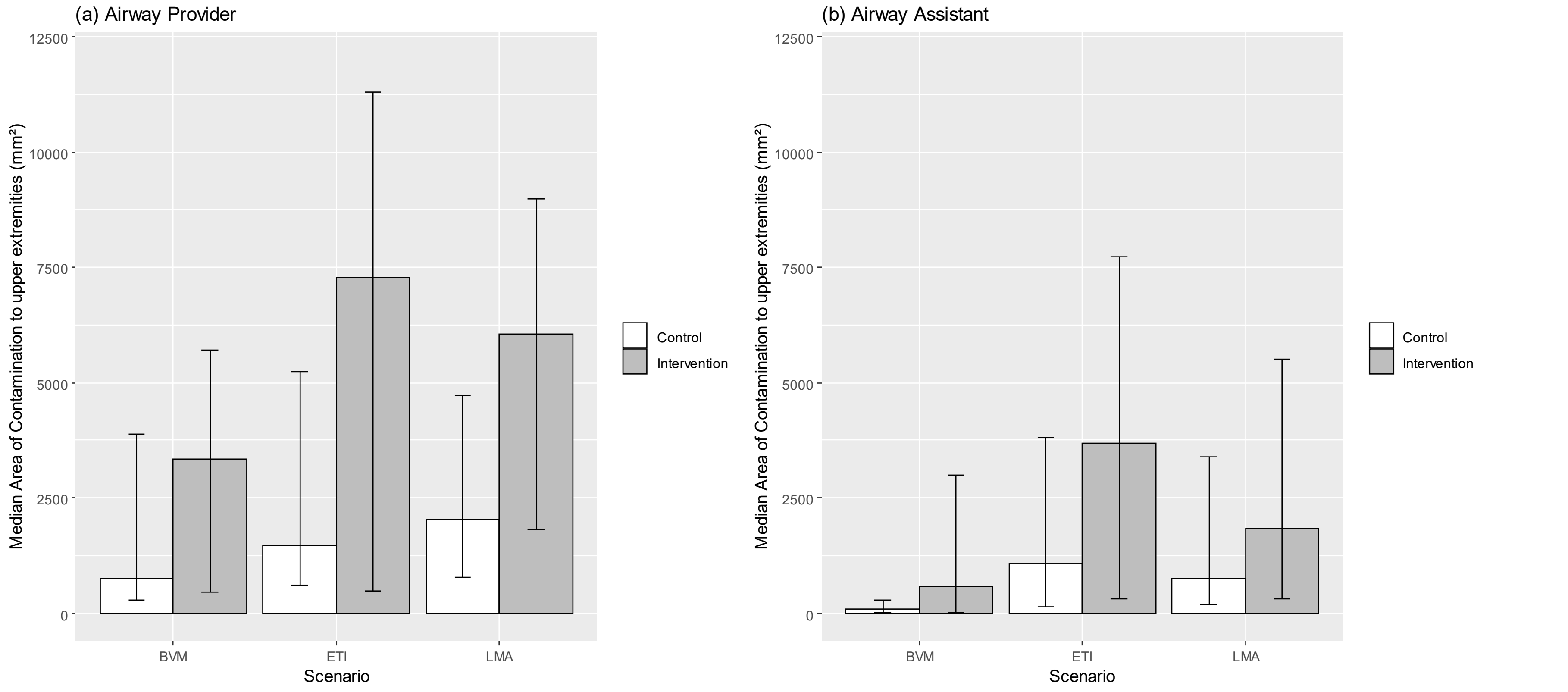

Error bar represents interquartile range

© 2023 Cheng A et al. *JAMA Network Open.*

eFigure 4: Median Area of contamination to torso (airway providers and assistants)

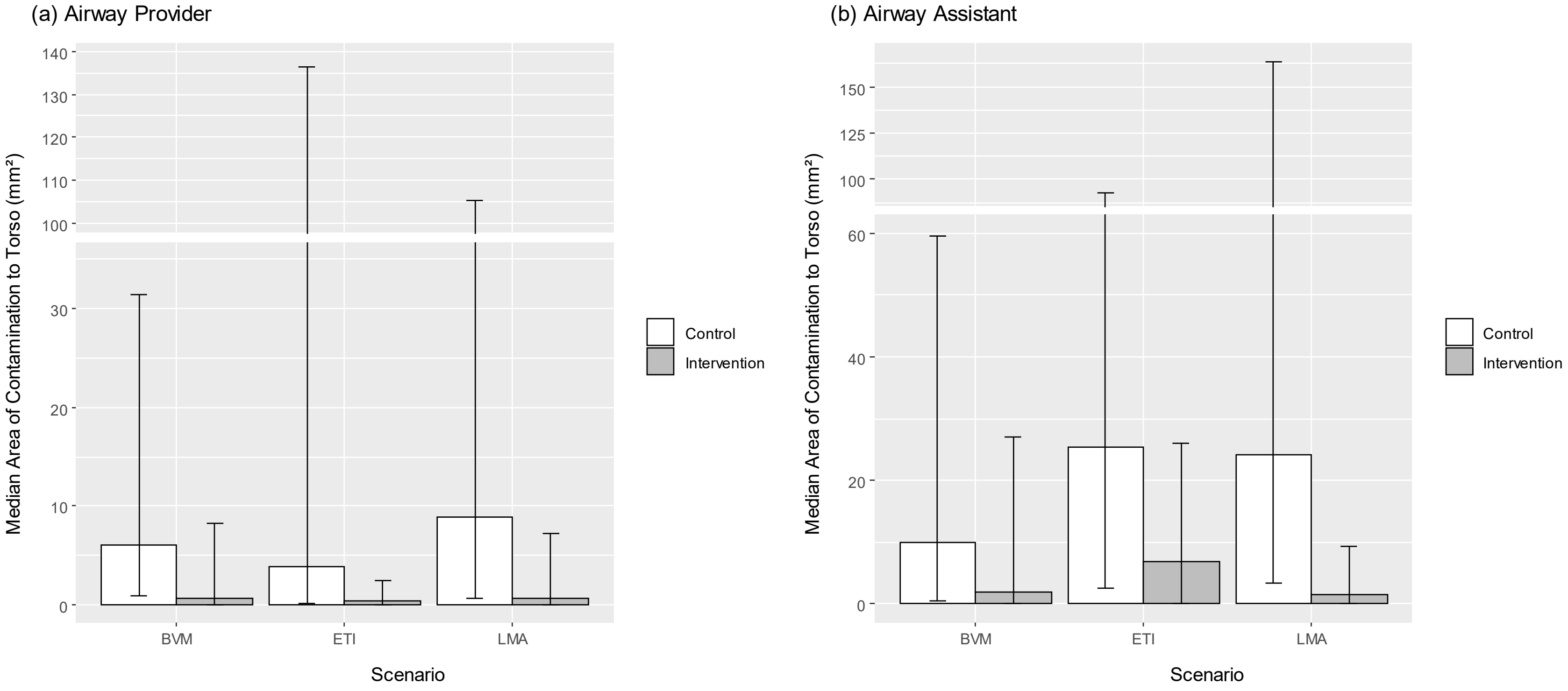

Error bar represents interquartile range

© 2023 Cheng A et al. *JAMA Network Open.*

eFigure 5: Proportion of participants with contamination to facial area (airway providers and assistants)

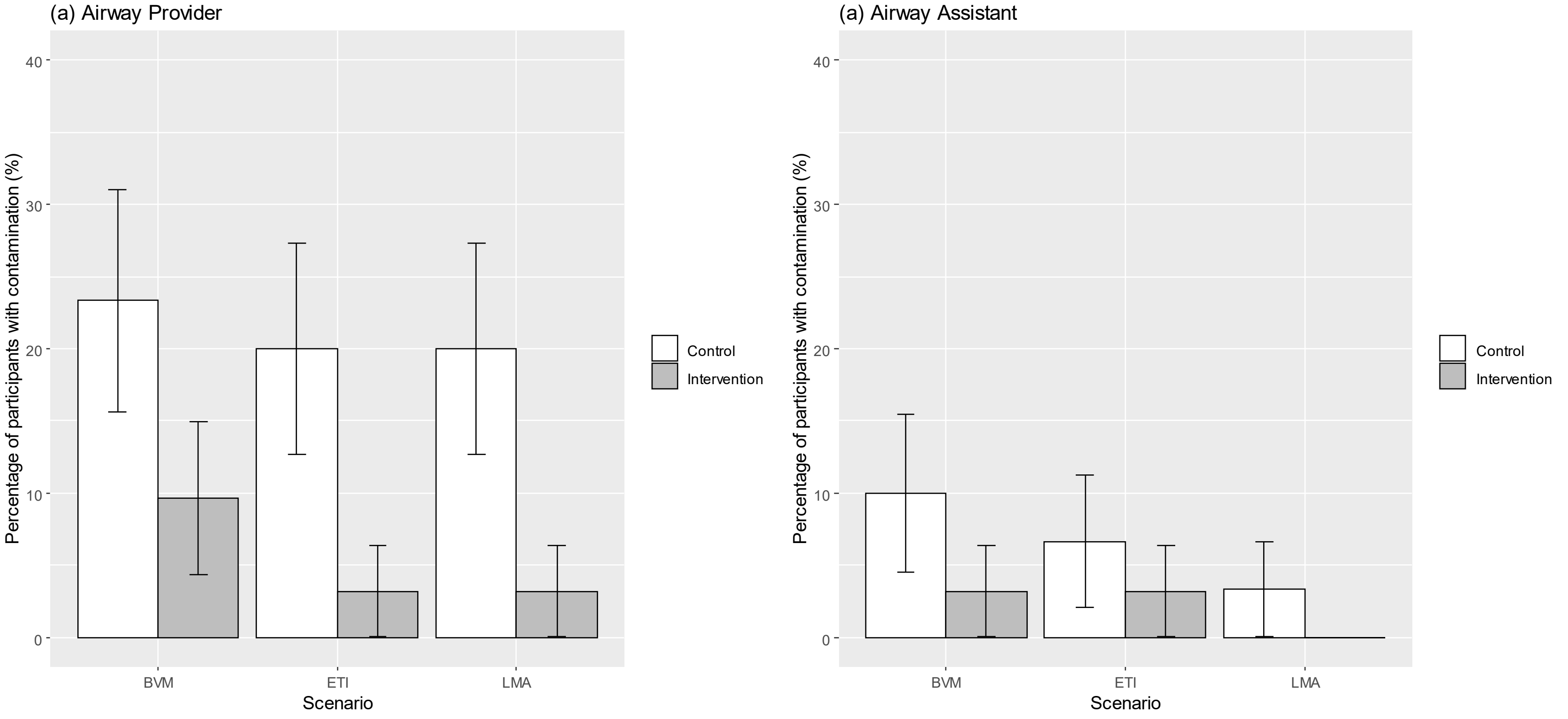

More than 75% of the participants have 0 AOC to the facial area. Therefore, the percentage of participants have > $1mm^2$ were presented in the figure.
Error bar represents ± 1 standard error (SE)

$SE = \sqrt{\frac{p\,(1-p)}{n}}$, where p is the proportion of participants with contamination and n is the sample size for each group.

© 2023 Cheng A et al. *JAMA Network Open.*

eTable 1: Bag Valve Mask Training Checklist

Objective: Ventilate with a bag-mask HEPA viral filter with a tight seal using the two-person technique

	1st Round Done? Y/N	2nd Round Done? Y/N
Insert the BVM, and the HEPA filter into the box through one of the openings		
Securely attach the HEPA viral filter to the ventilation device by placing it between the bag and the mask within the box		
Insert the oxygen tubing through the small semi-circular access ports at the front panel of the box, and connect it to the oxygen source		
Insert the yankauer suction equipment inside the box through one of the openings at the head		
Insert the suction tubing through the small semi-circular access ports at the head of the box and connect it to the yankauer		
Optimize the height of the stretcher according to the airway provider's comfort		
Ventilate with the bag-mask by using the two-person technique where:<br>- Provider standing at the front panel of the box ensures adequate seal<br>- The other provider standing at the side of the box ventilates		

© 2023 Cheng A et al. *JAMA Network Open.*

eTable 2: Endotracheal Intubation Training Checklist

Objective: Intubate with video-laryngoscope and ventilate with a bag-mask-HEPA viral filter.

	**1st Round Done? Y/N**	**2nd Round Done? Y/N**
Ventilate patient using a BVM with a HEPA filter in place, preferentially with the two-person technique		
Order appropriate RSI medication for the weight and condition of the patient		
Optimize the height of the stretcher according to the airway provider's comfort		
Prepare the equipment for the intubation:<br>- Select the appropriate blade for the VDL<br>- Select the appropriately sized cuffed ET tube, and lubricate it<br>- Select the stylet and insert it inside the ETT<br>- Select the syringe, connect it to the cuff of the ETT and test the cuff<br>- Choose a colorimetric capnography<br>- Prepare the tape to secure the tube		
Place all the equipment inside the box at once		
Intubate after administration of RSI medication		
Remove the stylet following intubation and inflate the cuff immediately without delay		
Connect the capnography device to the ETT, the HEPA filter to the capnography device, and the BVM to the HEPA filter		
Ventilate at 20-30/min		
Secure the tube in place		

© 2023 Cheng A et al. *JAMA Network Open.*

eTable 3: Laryngeal Mask Airway Training Checklist

Objective: Insert LMA and ventilate with a bag-mask-HEPA viral filter.

	**1st Round Done? Y/N**	**2nd Round Done? Y/N**
Ventilate the patient using a BVM with a HEPA filter in place, preferentially with the two-person technique		
Order appropriate RSI medication for the weight and condition of the patient		
Optimize the height of the stretcher according to the airway provider's comfort		
Prepare the equipment for the LMA insertion:<br>- Select the appropriate LMA size and lubricate it<br>- Select the syringe, connect it to the cuff of the LMA, and test the cuff<br>- Choose a colorimetric capnography<br>- Prepare the tape to secure the tube		
Place all the equipment inside the box at once		
Insert the LMA after administration of RSI medication		
Inflate the cuff immediately		
Connect the capnography device to the LMA, the HEPA filter to the capnography device, and the BVM to the HEPA filter		
Ventilate at 20-30/min		
Secure the tube in place		

eTable 4: Scenario – Bag Valve Mask Ventilation

Case Introduction:

- You are working at the Emergency Department and asked to reassess this 13y.o. boy. The patient is diagnosed with pneumonia and the COVID-19 viral test (nucleic acid) from 2 days ago was confirmed positive.
- You are asked to don the PPE and provide respiratory support with bag-valve-mask ventilation.
- You are not expected to secure a definitive airway; however, you may order RSI meds if you choose. Bag the patient till the end of the scenario.
- (If intervention group) An aerosol box has been placed
- The patient has IV fluid (D5NS) running at maintenance, is connected to patient monitor and weighs 40kg.

Other History:

- Coughing and fever for 3 days, worsening 2 days ago and respiratory distress developed today
- Close contact with family member who is diagnosed with COVID-19
- COVID test positive
- PMH: Previously healthy, no known allergy

Important Pre-simulation briefing points:

- The patient's chest will not rise despite excellent ventilation.
- Remind the participant to use oxygen source 1 only to manage the patient. Participants should ignore the noise caused by oxygen source 2.
- Airway assistant can leave the patient to prepare the equipment
- The ER nurse (confederate) will prepare and give medication.
- The ER nurse (confederate) will not assist in airway and breathing management, if RSI medication is ordered
- You are permitted to adjust the height of the bed
- Please bag at a rate of 20-30 ventilations / min (2020 AHA ECC guideline)

© 2023 Cheng A et al. *JAMA Network Open.*

Scenario Stage	Patient condition	Simulator Parameters	Expected Intervention	Confederate Roles
**Respiratory Failure (5 min)**	**History**<br>• 13-year-old boy (40 kg)<br>• PMH: unknown<br>• Acute Respiratory symptoms for 3 days, worsening today<br>• Confirmed COVID-19 Positive<br><br>**Condition:**<br>• Responsive to pain stimulation only<br>• Decreased respiratory rate and effort<br>• Skin color cyanotic<br><br>**Physical Exam**<br>• T 38.0, HR 150, RR 8, SpO2 90% (oxygen mask non-rebreather 100% O2)<br>• Monitor: sinus tachycardia<br>• CNS: responsive to pain stimulation<br>• CVS: pulse (+), CRT 2 sec, heart sound normal, no murmur<br>• Resp: decreased rate and effort, no crackles	**Vitals**<br>• T 38.0<br>• HR 150<br>• RR 8<br>• SpO_2 90% (100% on oxygen)<br>• BP 100/70<br><br>**Condition**<br>• Eyes closed<br>• Palpable pulse<br>• CRT 2 sec<br><br>**Rhythm**<br>• Sinus rhythm<br><br>**Moulage**<br>• Dress in shorts/shoes<br><br>**O2 source # 2:**<br>O2 flow set as 8L/min<br><br>**Facilitator bag at a rate of 8**	**PPE donning completed**<br>**Assess A-B-C**<br>**Airway**<br>• No airway obstructions<br>• Bag-Valve-Mask – good chest rises and fall (information provided the confederate)<br><br>**Breathing**<br>• Continue Bag-Valve-Mask at a rate of 20-30 / min<br>• Continuously monitor SpO2<br><br>**Circulation**<br>• Reassess BP, CRT, HR continuously<br><br>**(If intervention group) Hand equipment inside the aerosol box**<br><br>Connect the HEPA filter with the mask the anesthesia bag<br><br>**Continue to support breathing with bag-valve-mask ventilation for until the end of the scenario (4 min)**	**Confederate will**<br>• Connect patient to monitor<br>• Simulate drawing up meds if ordered but will not administer them.<br>• Correct the error, if an incorrect dose of medication is ordered.<br><br>**Confederate will NOT**<br>• Offer information about vital signs unless necessary<br>• Provide airway/respiratory support to the patient

		**breaths per minute to simulate patient's respiration.**	**Call for help and further support**<br><br>**Considering establishing advanced airway and ordering RSI medication**<br>• Lidocaine (40mg, 1mg/kg)<br>• Ketamine (40 mg, 1mg/kg)<br>• Succinylcholine (60mg, 1-2mg/kg)	

eTable 5: Scenario – Endotracheal Intubation

Case Introduction (Adolescent):

- You are working at the Emergency Department and asked to reassess this 14 y.o. boy who was admitted today. The patient is diagnosed with pneumonia and the COVID-19 viral test (nucleic acid) from 2 days ago was confirmed positive. The patient has deteriorated in the ED
- You are asked to provide respiratory support by inserting an ETT. You will order RSI meds, pre-oxygenate and bag-valve-mask ventilate the patient and perform the intubation with video laryngoscopy. Continue to bag the patient until the end of the scenario.
- (If intervention group) An aerosol box has been placed
- The patient has IV fluid (D5NS) running at maintenance, is connected to a patient monitor and weighs 50kg.

Other History:

- Coughing and fever for 3 days, worsening 2 days ago and respiratory distress developed today
- Close contact with family member who is diagnosed with COVID-19
- COVID test positive
- PMH: Previously healthy, no known allergy

Important Pre-simulation briefing points:

- The patient's chest will not rise despite excellent ventilation.
- Remind the participant to use oxygen source 1 only to manage the patient. Participants should ignore the noise caused by oxygen source 2.
- Airway assistant can leave the patient to prepare the equipment
- The ER nurse (confederate) will prepare and give RSI medications.
- The ER nurse (confederate) will not assist in airway and breathing management
- You are permitted to adjust the height of the bed
- Please bag at a rate of 20-30 ventilations / min (2020 AHA ECC guideline)

© 2023 Cheng A et al. *JAMA Network Open.*

Scenario Stage	Patient condition	Simulator Parameters	Expected Intervention	Confederate Roles
**Respiratory Failure (5 min)**	**History**<br>• 14-year-old boy (50 kg)<br>• PMH: unknown<br>• Acute Respiratory symptoms for 3 days, worsening today<br>• Confirmed COVID-19 Positive<br><br>**Condition:**<br>• Responsive to pain stimulation only<br>• Decreased respiratory rate and effort<br>• Skin color cyanotic<br><br>**Physical Exam**<br>• T 38.0, HR 150, RR 8, SpO2 88%<br>• Monitor: sinus tachycardia<br>• CNS: responsive to pain stimulation<br>• CVS: pulse (+), CRT 2 sec, heart sound normal, no murmur<br>• Resp: decreased rate and effort, no crackles	**Vitals**<br>• T 38.0<br>• HR 150<br>• RR 8<br>• SpO_2 88%<br>• BP 100/70<br><br>**Condition**<br>• Eyes closed<br>• Palpable pulse<br>• CRT 2 sec<br><br>**Rhythm**<br>• Sinus rhythm<br><br>**O2 source # 2:**<br>Initial: 8/L<br><br>Once patient paralyzed, RR =0 on monitor.	**PPE donning completed**<br>**Assess A-B-C**<br>**Airway**<br>• No airway obstructions<br>• Good chest rise and fall with BVM (information provided by confederate)<br><br>**Breathing**<br>• Continue Bag-Valve-Mask at a rate of 20-30 / min<br>• Continuously monitor SpO2<br><br>**Circulation**<br>• Reassess BP, CRT, HR continuously<br><br>**(If intervention group) Hand video laryngoscope inside the aerosol box**<br><br>**Continue to support breathing with bag-valve-mask ventilation until RSI medication is given.**<br><br>**Order medication before inserting ETT**<br>• Ketamine (50 mg, 1mg/kg)	**Confederate will**<br>• Prepare RSI medication and give the meds (but not before the 2 minute mark).<br>• Correct the error if an incorrect dose is ordered.<br><br>**Confederate will NOT**<br>• Offer information about vital signs unless necessary<br>• Provide airway/respiratory support to the patient

			• Succinylcholine (50-100mg, 1-2mg/kg)<br><br>**Insert the ETT with video laryngoscope**<br><br>**Inflate the cuff and checking for placement of the tube using colored CO2 detector**<br><br>**Continue to bag the patient until the end of the scenario (5 min)**	

© 2023 Cheng A et al. *JAMA Network Open.*

eTable 6: Scenario – Laryngeal Mask Airway Insertion

Case Introduction (Adolescent):

- You are working at the Emergency Department and asked to reassess this 14 y.o boy admitted today. The patient is diagnosed with pneumonia and the COVID-19 viral test (nucleic acid) confirmed positive from 2 days ago. The patient has deteriorated.
- You are asked to don the PPE and provide respiratory support by inserting an LMA. You will bag-valve-mask ventilate the patient, order RSI meds and insert the LMA. Continue to bag the patient till the end of the scenario.
- (If intervention group) An aerosol box has been placed
- The patient has IV fluid (D5NS) running at maintenance, is connected to a patient monitor and his weight is 45 kg.

Other History:

- Coughing and fever for 3 days, worsening 2 days ago and respiratory distress developed today
- Close contact with family member who is diagnosed with COVID-19
- COVID test positive
- PMH: Previously healthy, no known allergy

Important Pre-simulation briefing points:

- The patient's chest will not rise despite excellent ventilation.
- Remind the participant to use oxygen source 1 only to manage the patient. Participants should ignore the noise caused by oxygen source 2.
- Airway assistant can leave the patient to prepare equipment if needed
- The ER nurse (confederate) will prepare and give medication, if RSI meds are ordered
- The ER nurse (confederate) will not assist in airway and breathing management
- You are permitted to adjust the height of the bed
- Please bag at a rate of 20-30 ventilations / min (2020 AHA ECC guideline)

Scenario Stage	Patient condition	Simulator Parameters	Expected Intervention	Confederate Roles
**Respiratory Failure (5 min)**	**History**<br>• 14-year-old boy (45 kg)<br>• PMH: unknown<br>• Acute Respiratory symptoms for 3 days, worsening today<br>• Confirmed COVID-19 Positive<br><br>**Condition:**<br>• Responsive to pain stimulation only<br>• Decreased respiratory rate and effort<br>• Skin color cyanotic<br><br>**Physical Exam**<br>• T 38.0, HR 150, RR 6, SpO2 88%<br>• Monitor: sinus tachycardia<br>• CNS: responsive to pain stimulation<br>• CVS: pulse (+), CRT 2 sec, heart sound normal, no murmur<br>• Resp: decreased rate and effort, no crackles	**Vitals**<br>• T 38.0<br>• HR 150<br>• RR 8<br>• SpO_2 88%<br>• BP 100/70<br><br>**Condition**<br>• Eyes closed<br>• Palpable pulse<br>• CRT 2 sec<br><br>**Rhythm**<br>• Sinus rhythm<br><br>**Moulage**<br>• Dress in shorts/shoes<br><br>**O2 source # 2:**<br>O2 flow set as 8 L/min<br><br>O2 flow off once the patient is paralyzed	**PPE donning completed**<br>**Assess A-B-C**<br>**Airway**<br>• No airway obstructions<br>• Good chest rise and fall with BVM (information provided the confederate)<br><br>**Breathing**<br>• Continue Bag-Valve-Mask at a rate of 20-30 / min<br>• Continuously monitor SpO2<br><br>**Circulation**<br>• Reassess BP, CRT, HR continuously<br><br>**(If intervention group) Hand airway equipment inside the aerosol box**<br><br>**Continue to support breathing with bag-valve-mask ventilation until RSI medication is given.**<br><br>**Order and administer medication before inserting LMA**<br>• Ketamine (45mg, 1mg/kg)<br>• Succinylcholine (60mg, 1-2mg/kg)	**Confederate will**<br>• Connect patient to monitor<br>• Prepare RSI medication and give the meds (but not before the 2 minute mark).<br>• Correct the error if an incorrect dose is ordered.<br><br>**Confederate will NOT**<br>• Offer information about vital signs unless necessary<br>• Provide airway/respiratory support to the patient

© 2023 Cheng A et al. *JAMA Network Open.*

			**Inserting the LMA**<br><br>**Inflating the cuff and checking for leak**<br><br>**Continue to bag the patient until the end of the scenario (5 min)**	

eTable 7: Pre-doffing area of contamination of airway providers

Mean (95%CI)		Control (mm^2) [a] mean of log-AOC	Intervention (mm^2) [a] Mean of log-AOC	Mean difference of log-AOC (mm^2)	P-value	Control (mm^2) [b] Geometric mean	Intervention (mm^2) [b] Geometric mean	Difference [c]
Overall effect	Upper extremity	6.52 (4.37, 8.67)	7.02 (4.87, 9.17)	0.50 (0.07, 0.93)	0.02	680.7 (78.3, 5817.8)	1124.3 (129.8, 9596.5)	65.0 (7.1, 154.6) %
	Torso	2.39 (1.18, 3.58)	0.90 (-0.31, 2.08)	-1.49 (-1.99, -0.99)	<0.001	9.9 (2.3, 34.7)	1.4 (-0.3, 7.0)	-77.5 (-86.3, -62.9) %
	Face	1.00 (0.12, 1.85)	0.06 (-0.81, 0.92)	-0.93 (-1.39, -0.47)	<0.001	1.7 (0.1, 5.4)	0.1 (-0.6, 1.5)	-60.7 (-75.2, -37.8) %
BVM	Upper extremity	6.21 (5.24, 9.19)	6.93 (5.97, 7.89)	0.72 (-0.64, 2.09)	0.30	498.8 (187.7, 1322.6)	1026.1 (393.0, 2676.4)	105.5 (-47.5, 705.6) %
	Torso	2.29 (1.62, 2.96)	1.25 (0.59, 1.91)	-1.04 (-1.98, -0.10)	0.03	8.9 (4.1, 18.4)	2.5 (0.1, 6.2)	-64.6 (-86.2, -9.6) %
	Face	1.27 (0.58, 1.97)	0.35 (-0.33, 1.03)	-0.92 (-1.89, 0.05)	0.06	2.6 (0.8, 6.2)	0.4 (0.0, 5.6)	-60.2 (-85.0, 5.1) %
ETI	Upper extremity	7.30 (6.66, 7.93)	7.94 (7.31, 5.57)	0.64 (-0.25, 1.54)	0.15	1481.1 (781.7, 2805.2)	2821.7 (1505.3, 5288.4)	90.4 (-22.2, 366.3) %
	Torso	2.65 (1.93, 3.38)	0.89 (0.18, 1.60)	-1.76 (-2.77, -0.74)	0.001	13.2 (5.8, 28.4)	1.4 (1.1, 15.1)	-82.7 (-93.7, -52.3) %
	Face	0.98 (0.42, 1.55)	0.12 (-0.43, 0.68)	-0.86 (-1.65, -0.07)	0.03	1.7 (0.5, 3.7)	0.1 (0.1, 4.2)	-57.8 (-80.9, -6.6) %
LMA	Upper extremity	7.62 (6.95, 8.28)	7.92 (7.26, 8.57)	0.30 (-0.64, 1.23)	0.52	2044.0 (1049.4, 3980.5)	2752.8 (1428.8, 5302.7)	34.6 (-47.1, 242.8) %
	Torso	2.74 (2.03, 3.46)	1.27 (0.56, 1.97)	-1.47 (-2.48, -0.47)	0.004	14.5 (6.6, 30.9)	2.6 (0.8, 6.2)	-77.1 (-91.6, -37.4) %
	Face	1.08 (0.47, 1.70)	0.15 (-0.45, 0.76)	-0.93 (-1.79, -0.06)	0.04	2.0 (0.6, 4.5)	0.2 (0.1, 5.0)	-60.5 (-83.3, -6.2) %

CI: confidence interval; AOC: Area of contamination); ln: natural log; BVM: Bag-valve-mask ventilation; ETI: Endotracheal intubation; LMA: Laryngeal mask airway.
Numbers in the brackets are 95% confidence interval; All area of contaminations were measured as mm^2
a. Arithmetic mean of log-AOC. The log-AOC is calculated as natural log of (AOC+1)
b. Geometric mean of AOC calculated by exponentiating the mean of log-AOC, then subtracting 1. Therefore, the 95%CIs are not symmetric.
c. Difference calculated by exponentiating the mean difference of log-AOC and subtracting 1, then multiplying by 100%. For example, the relative difference of Torso contamination reported in the overall model (-77.5%) is interpreted as, compared to control group, the use of aerosol box is associated with a 77.5% lower geometric mean of AOC.

© 2023 Cheng A et al. *JAMA Network Open.*

eTable 8: Pre-doffing area of contamination of airway assistants

Mean 95%CI		Control (mm²) [a] mean of log-AOC	Intervention (mm²) [a] Mean of log-AOC	Mean difference of log-AOC (mm²)	P-value	Control (mm²) [b] Geometric mean	Intervention (mm²) [b] Geometric mean	Difference [c]
Overall effect	Upper extremity	4.81 (2.14, 7.47)	5.69 (3.02, 8.35)	0.88 (0.29, 1.48)	0.004	121.6 (7.5, 1748.5)	296.4 (19.6, 4234.4)	142.4 (34.0, 339.2) %
	Torso	2.53 (0.91, 4.16)	1.30 (-0.33, 2.92)	-1.23 (-1.69, -0.77)	<0.001	11.6 (1.5, 63.2)	2.7 (-0.3, 17.6)	-70.9 (-81.6, -54.1) %
	Face	0.12 (-0.08, 0.32)	0.09 (-0.11, 0.29)	-0.03 (-0.23, 0.17)	0.78	0.1 (-0.1, 0.4)	0.1 (-0.1, 0.3)	-2.8 (-20.1, 18.4) %)
BVM	Upper extremity	4.16 (3.13, 5.20)	5.38 (4.37, 6.40)	1.22 (-0.23, 2.67)	0.10	63.6 (22.0, 180.6)	217.6 (78.0, 603.8)	238.6 (-20.6, 134.4) %
	Torso	2.38 (1.67, 3.08)	1.68 (0.99, 2.37)	-0.70 (-1.68, 0.28)	0.16	9.8 (4.4, 20.9)	4.4 (1.7, 9.7)	-50.4 (-81.4, 33.1) %
	Face	0.29 (-0.05, 0.64)	0.17 (-0.16, 0.51)	-0.12 (-0.60, 0.36)	0.62	0.3 (0.0, 0.9)	0.2 (-0.2, 0.6)	-11.3 (-45.2, 43.7) %
ETI	Upper extremity	6.19 (5.23, 7.14)	7.06 (6.12, 8.00)	0.87 (-0.46, 2.21)	0.20	487.0 (187.0, 1265.3)	1165.1 (455.4, 2978.3)	138.9 (-37.2, 810.3) %
	Torso	2.91 (2.21, 3.60)	1.94 (1.25, 2.63)	-0.96 (-1.94, 0.01)	0.05	17.3 (8.1, 35.8)	6.0 (2.5, 12.8)	-61.9 (-85.7, 1.3) %
	Face	0.10 (-0.17, 0.37)	0.16 (-0.10, 0.42)	0.06 (-0.31, 0.43)	0.75	0.1 (-0.2, 0.4)	0.2 (-0.4, 0.4)	6.1 (-27.0, 54.3) %
LMA	Upper extremity	6.02 (5.06, 6.98)	6.86 (5.92, 7.81)	0.84 (-0.50, 2.19)	0.22	412.8 (157.5, 1079.2)	959.6 (372.8, 2468.0)	132.1 (-39.5, 791.9) %
	Torso	3.27 (2.52, 4.02)	1.46 (0.72, 2.20)	-1.81 (-2.86, -0.75)	0.001	25.4 (11.4, 54.9)	3.3 (1.1, 8.0)	-83.6 (-94.3, -53.1) %
	Face	0.01 (0.00, 0.03)	0.00 (-0.01, 0.04)	-0.01 (-0.04, 0.01)	0.31	-0.01 (-0.03, 0.01)	0.00 (-0.01, 0.04)	-1.2 (-3.6, 1.2) %

CI: confidence interval; AOC: Area of contamination); ln: natural log; BVM: Bag-valve-mask ventilation; ETI: Endotracheal intubation; LMA: Laryngeal mask airway.
Numbers in the brackets are 95% confidence interval; All area of contaminations were measured as mm²
a. Arithmetic mean of log-AOC. The log-AOC is calculated as natural log of (AOC+1)
b. Geometric mean of AOC calculated by exponentiating the mean of log-AOC, then subtracting 1. Therefore, the 95%CIs are not symmetric.
c. Difference calculated by exponentiating the mean difference of log-AOC and subtracting 1, then multiplying by 100%. For example, the relative difference of Torso contamination reported in the overall model (-70.9%) is interpreted as, compared to control group, the use of aerosol box is associated with a 70.9% lower geometric mean of AOC.

© 2023 Cheng A et al. *JAMA Network Open.*

eTable 9: Proportion of participants with contamination (post-doffing)

Scenario	Upper Extremities			Torso			Face			Total		
	Control (n=60)	Interven -tion (n=62)	p-value	Control (n=60)	Interven-tion (n=62)	p-value	Control (n=60)	Interven-tion (n=62)	p-value	Control (n=60)	Interven-tion (n=62)	p-value
BVM	0 (0.0%)	0 (0.0%)	> 0.99	0 (0.0%)	0 (0.0%)	> 0.99	1 (3.3%)	0 (0.0%)	0.49	1 (3.3%)	0 (0.0%)	0.49
ETI	0 (0.0%)	2 (3.2%)	0.50	0 (0.0%)	2 (3.2%)	0.50	1 (3.3%)	0 (0.0%)	0.49	1 (3.3%)	2 (3.2%)	0.58
LMA	0 (0.0%)	1 (1.6%)	> 0.99	0 (0.0%)	0 (0.0%)	> 0.99	0 (0.0%)	0 (0.0%)	> 0.99	0 (0.0%)	1 (1.6%)	> 0.99

© 2023 Cheng A et al. *JAMA Network Open.*

eTable 10: Factors associated with the AOC deposited on healthcare providers (pre-doffing)

		Upper Extremities		Torso and Face	
		Adjusted coefficient (95% CI)	p-value	Adjusted coefficient (95% CI)	p-value
**Intercept**		4.61 (1.51, 7.72)	0.005	2.60 (0.08, 5.12)	0.05
**Group**	Control	0 [reference]		0 [reference]	
	Intervention	0.75 (0.01, 1.48)	0.05	-1.40 (-1.99, -0.80)	<0.001
**Role**	Provider	0 [reference]		0 [reference]	
	Assistant	-1.36 (-2.09, -0.62)	<0.001	0.22 (0-.37, 0.81)	0.47
**Procedure**	BVM	0 [reference]		0 [reference]	
	ETI	1.44 (1.06, 1.82)	<0.001	0.06 (-0.30, 0.42))	0.75
	LMA	1.43 (1.05, 1.81)	<0.001	0.15 (-0.21, 0.52))	0.40
**BSA (m^2)**		0.76 (-0.92, 2.44)	0.38	0.06 (-1.30, 1.42)	0.93

BVM: Bag-valve-mask ventilation; ETI: endotracheal intubation; LMA: laryngeal mask airway insertion 2

Dependent variable: log-AOC calculated as ln (AOC +1)

The coefficient presented in the tables are the differences of log-means.

© 2023 Cheng A et al. *JAMA Network Open.*
