# Supplementary material for: Aerosol Box Use in Reducing Health Care Worker Contamination During Airway Procedures (AIRWAY Study): A Simulation-Based Randomized Clinical Trial
Source: JAMA Netw Open. 2023 Apr 12;6(4):e237894. doi: 10.1001/jamanetworkopen.2023.7894 (PMC10099073; doi:10.1001/jamanetworkopen.2023.7894)
Supplement: Supplement 2. — Trial Protocol [file jamanetwopen-e237894-s002.pdf]

1.0 THE NEED FOR A TRIAL

1.1 Problem to be addressed.

To date, the Coronavirus Disease 2019 (COVID-19) pandemic, caused by severe acute respiratory syndrome coronavirus 2 (SARS-CoV-2), has resulted in over 36 million documented cases and over 1 million deaths[1]. Patients infected with SARS-CoV-2 experience unusually high rates of critical illness requiring advanced airway management and intensive care unit admission[2-8]. Data from the epicenter of the COVID-19 outbreak in Wuhan, China, indicate that 2.3 to 3.2% of patients with COVID-19 required endotracheal intubation (ETI) and invasive ventilation at some point during their illness[2, 4]. The overwhelming scale of the pandemic and severity of disease presents a serious threat to the health of frontline workers. In Italy, 9% of over 22,000 SARS-CoV-2 cases were healthcare providers (HCP)[3]. Aerosol-generating medical procedures (AGMPs), such as bag-valve-mask (BVM) ventilation, laryngeal mask airway insertion (LMA), and ETI are commonly required for critically ill COVID-19 patients. AGMPs produce airborne particles, contributing to the disproportionately high risk of infection amongst HCPs working in acute care areas[9, 10]. Strategies to mitigate HCPs exposure to and infection from COVID-19 during AGMPs are required to maintain the integrity of the healthcare workforce during the pandemic.

To minimize HCP exposure during high-risk AGMPs, an aerosol box has been developed to place over the head of the patient, shielding the provider's face from aerosols[11]. This invention, and various different adaptations, have been implemented by hospitals around the world for airway management in COVID-19 patients[12-16]. During the COVID-19 pandemic, members of our research team have developed and utilized an adapted version of the aerosol box for the management of COVID-19 patients undergoing AGMPs (Figure 1)[17]. Little is known about the effectiveness of the aerosol box during BVM ventilation, LMA insertion or ETI. Several small studies done in simulated environments with manikin heads enclosed by aerosol boxes demonstrate variable containment of particles aerosolized by a simulated cough[12, 14, 16, 18, 19], but none of these studies incorporated wall suction as a means to provide negative airflow. A more recent study demonstrated significantly reduced levels of airborne particles measured at the laryngoscopist's head when using an aerosol box with wall suction, compared to an aerosol box without wall suction[20]. Despite this promising data, there has been concern about the impact of aerosol box use on procedural performance, which may potentially have a negative effect on patient outcomes. Amongst a group of 12 anesthesiologists, aerosol box use was associated with longer intubation times and lower first-pass success rates when attempting intubation on an adult manikin head[21]. The main limitations of this study were that participants received only very brief training, and did not have an airway assistant to support them during ETI. To date, there have been no single or multicenter studies assessing the use of an aerosol box with trained airway teams (i.e. airway provider and airway assistant). Studies have focused primarily on the task of intubation, without any evidence describing the impact on performance of LMA insertion or BVM ventilation. Furthermore, no studies have quantified the degree of contamination resulting from AGMPs with and/or without the use of an aerosol box while concurrently measuring clinically important performance outcomes (eg. time to intubation or LMA insertion).

Our study will provide evidence to: (a) determine if aerosol boxes are effective in reducing provider and environmental contamination during performance of AGMPs by a trained airway team; (b) evaluate if using an aerosol box adversely affects time to completion and first pass success rates for ETI and LMA insertion; and (c) describe the patterns of contamination after commonly performed AGMPs. Our *long-term goal* is to provide empiric evidence to enhance HCP safety during AGMPs while delivering high quality care. This will be accomplished by creating clinical scenarios within a simulated clinical environment, as conducting a similar study on real COVID-19 patients would be fraught with challenges and risks[22, 23]. By using manikins as surrogate patients, we will quantitatively measure HCP and environmental contamination after performing AGMPs. Measuring time to achieve

successful ETI and LMA insertion and first-pass success rates provide clinically relevant parameters that can be balanced against the degree of HCP contamination to inform clinical practice and potential changes to institutional policy.

Figure 1 – Aerosol Box

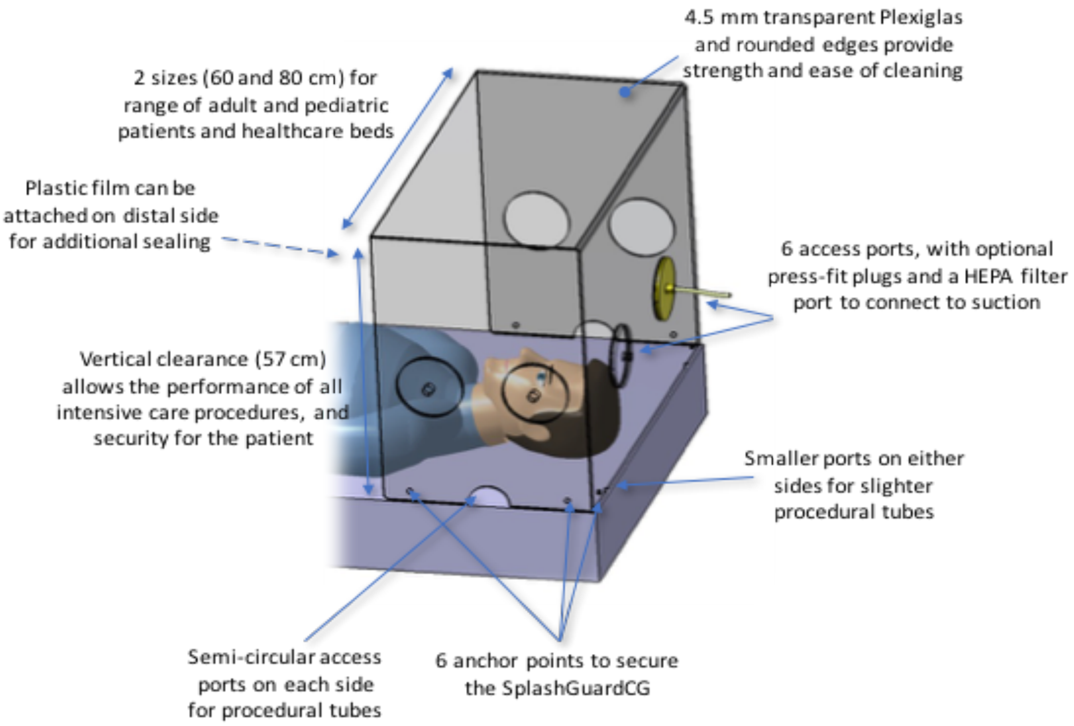

1.2 Principal research questions.

The *primary aim* of this study is to evaluate the effectiveness of aerosol box use, compared with no aerosol box use, for reducing HCP contamination during performance of AGMPs by a trained airway team during care of a simulated patients in respiratory failure due to COVID-19. We hypothesize that the use of an aerosol box, compared with no aerosol box use, will reduce HCP contamination caused by AGMPs during care of simulated patients in respiratory failure due to COVID-19.

The *secondary aim* is to determine if aerosol box use influences the time to successful completion and first-pass success rate for ETI and LMA insertion in trained airway teams. We hypothesize that there will be no significant differences in time to ETI and LMA insertion and first pass success rates when comparing airways teams using an aerosol box with teams not using an aerosol box.

1.3 Why is a trial needed now?

The second wave of the COVID-19 pandemic is upon us. Now, more than ever, it is critically important to identify strategies to protect healthcare workers from infection with SARS-CoV-2. Failure to do so may result in workforce shortages, which in turn will compromise patient safety. AGMPs are procedures that have the potential to create aerosols, or tiny particles suspended in the air that can contain pathogens such as viruses[10, 24]. Studies conducted during the prior SARS-CoV epidemic suggest that AGMPs play an important role in virus transmission via aerosols[10, 25, 26]. A systematic review identified BVM ventilation and ETI as AGMPs associated with highest risk of SARS-CoV transmission[10]. As SARS-CoV and SARS-CoV-2 are from the same family of Coronaviridae, this data raises significant concern regarding the role of AGMPs in nosocomial

transmission of SARS-CoV-2. Given the paucity of experimental data on nosocomial virus transmission and AGMPs, most guidelines for infection control are based expert opinion[24]. No studies to date have quantified the degree of HCP contamination resulting from AGMPs performed while caring for critically ill patients. Demonstrating reduced HCP contamination with use of the aerosol box during AGMPs can potentially enhance HCP safety, resulting in reduced workforce shortages due to illness.

1.4 How will the results of this trial be used?

Research assessing strategies to mitigate HCP risks of contamination is critical to reduce rates of HCP infection with SARS-CoV-2. Our study will be the first to supportive evidence for aerosol box use with trained airway teams performing AGMPs in patients with respiratory failure. Our study will also be the first to provide a quantitative measure of HCP contamination patterns (pre- and post-doffing) in conjunction with ETI and LMA insertion times. In combination, these metrics provide key information that will inform clinical practice, institutional airway management policies, and PPE protocols. Data from our study will be used to inform future clinical studies on real COVID-19 patients, or in other clinical contexts where AGMPs pose a health risk to HCPs. Existing partnerships with institutions within the International Network for Simulation-based Pediatric Innovation, Research and Education (INSPIRE network) will enable rapid dissemination of aerosol box training material across all continents. Given that aerosol boxes are both pragmatic and affordable, positive results from our study will inform clinical practice changes that will help mitigate healthcare provider risks and reduce morbidity and mortality from SARS-CoV-2.

1.5 Risks to participants.

There are no risks to the safety of participants involved in this trial.

2.0 THE PROPOSED TRIAL

2.1 Trial design.

We plan to conduct a prospective, randomized controlled trial at four INSPIRE network sites (Alberta Children's Hospital, Ste. Justine Hospital, and Children's Hospital of Los Angeles and The Hospital for Sick Children). Simulation-based research confers the advantage of answering research questions without risk of harm to HCPs or patients, which is particularly important when studying a disease process with high mortality[22]. Ethics approval has been submitted at all sites. Two participants will form an airway team, recruited to play the roles of airway provider and airway assistant for management of a simulated, critically ill COVID-19 patient. Participants will be randomized by team into either the control arm (i.e. no aerosol box) or the intervention arm (i.e. use of aerosol box) (Figure 2). Following randomization, all participants will view a short video orienting them to the simulated clinical environment. Intervention arm teams will view an additional video orienting them to the use of the aerosol box, and receive up to 15 minutes of hands-on training (see below for details). After orientation, teams will participate in three sequential simulation scenarios. The order of scenario delivery will be randomized to eliminate scenario order as a potential confounder. The location and nature of equipment, temperature and humidity within the resuscitation room will be standardized across all sites. A Laerdal Resusci-Anne manikin will be used as a simulated patient.

Figure 2 – Study Design

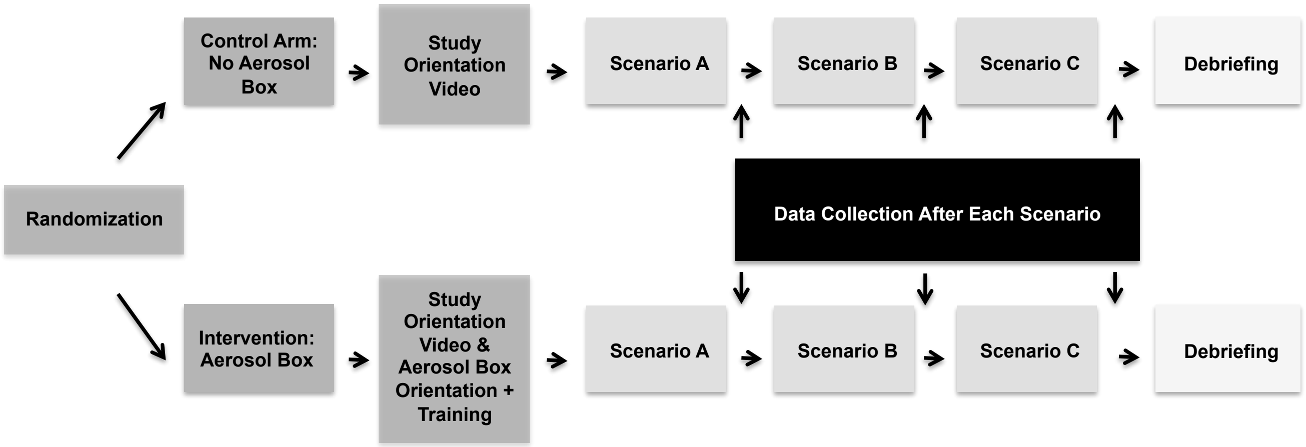

2.2 Trial interventions.

The aerosol box is a transparent, plastic cube covering the patient's head and shoulders, with circular access ports on the front of the box allowing for access to manage the airway (see budget). An additional four access holes (i.e. two on either side of the box) allow for airway assistants to access the patient airway (Figure 3). As our study includes only one airway assistant, the two holes that are not in use will be sealed shut during the study. The airway assistant will be directed to stand in a standardized position, to the immediate right of the airway provider. In a prior study, an aerosol box placed over the head of an adult volunteer demonstrated air leaks out of the box during exhalation and coughing that could be eliminated with the addition of a plastic drape[15]. For this reason, we have incorporated a plastic drape extending from the top of the box down to the patient's chest to prevent spread of aerosols. In another experiment, the addition of continuous wall suction to the aerosol box setup resulted in significantly decreased airborne particle exposure (for the airway provider) compared to aerosol box use without suction[20]. Given that wall suction is ready available and accessible in most acute care areas, our aerosol box setup will include wall suction, with suction tubing running into the aerosol box at one point along the bottom of the box near the head of the patient. Wall suction will be set at 200 mmHg, which is consistent with the pressure used in prior studies[20] and measured in the engineering lab of Dr. Aubin (co-applicant) to generate a negative airflow of approximately 50 L/min.

All participants (i.e. airway provider and airway assistant) randomized to the intervention arm will receive aerosol box training. A short video will orient them to the design of the aerosol box and include expert-modeled demonstration of strategies for optimal BVM ventilation, ETI, and LMA insertion using an aerosol box with a 2-person airway team. The training video will be shot in English and French to permit viewing across study sites in Alberta, Ontario and Quebec. After viewing the video, participants will work in pairs to practice all three procedures for a maximum of 15 minutes, providing them opportunity to coordinate their movements to optimize efficiency. After each procedure, they will receive feedback from a local airway and aerosol box expert (i.e. site investigator).

Figure 3 – Aerosol Box Setup

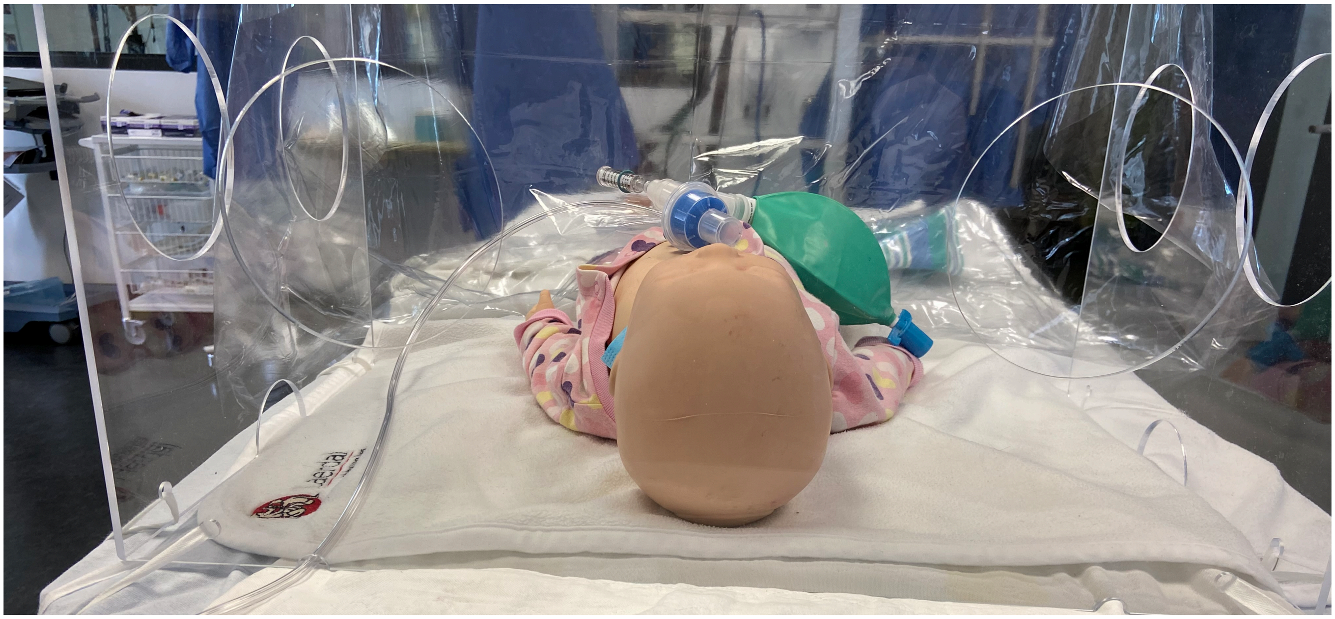

Simulating Aerosolization.

The amount of aersolization that occurs in real patients is highly variable and dependent upon many factors (e.g. viral load, method of aerosolization, etc.), making it impossible to exactly replicate aerosolization of SARS-CoV-2 virus in the simulated environment. In our study, we aim to create a model of aerosolization by standardizing various aspects of care (e.g. ventilation pressures, particle size and volume deposited, lung compliance), thus allowing us to make comparisons between different contexts (ie. aerosol box vs no box; BVM vs. LMA vs. ETI). To visualize aerosolization of particles, we will adapt methodology successfully utilized by members of our research team in studies evaluating aerosolization during airway management[9, 18]. GloGerm©™(Glo Germ Company, Moab, UT, USA) is a nontoxic, invisible fluorescent resin marker that illuminates when exposed to ultraviolet (UV) light. The SARS-CoV-2 virus requires a water and mucus envelope to spread, with the size of these virus-containing envelopes varying from larger droplets (>60 µm) to smaller airborne particles or infectious droplet nuclei (5-10 µm diameter)[24, 27]. With a particle size of approximately 1-5 µm, Glo Germ™ represents a reasonable surrogate for aerosolized SARS-CoV-2. AGMPs can generate aerosols in two ways: by mechanically inducing and dispersing aerosols, or by inducing the patient to cough to produce aerosols. Our study focuses on aerosols mechanically induced by three commonly performed AGMPs: BVM ventilation, LMA insertion, and ETI. Glo Germ™(0.5 mL) will be applied to the oropharynx and trachea of the manikin to simulate secretions. Glo Germ™ will be aerosolized from the mechanical pressures and airflow associated with bagging, or from manipulation of the airway, which is consistent with the current understanding of aerosolization mechanics in AGMPs[24]. Participants will titrate ventilation pressures to 20 cm H2O peak inspiratory pressure, and 5-6 cm H2O peak end expiratory pressure using a digital pressure manometer that provides real-time feedback. Pilot work done by our research team using the methods (and ventilation pressures) described above resulted in contamination on the hands, torso, face shield, and feet of airway providers during BVM ventilation. This suggests these methods are sufficient to produce a measurable amount of Glo Germ™ particles during manual ventilation[28].

Simulation Scenarios.

Prior to each scenario, all participants will donn personal protective equipment (PPE), consisting of: a gown, nitrile gloves, face shield, googles, and a N95 respirator (see budget). The brand, type and size of gowns and face shields will be standardized across all sites. PPE will be donned with a partner, guided by a standardized PPE donning checklist, and checked by a research assistant prior to the scenarios. All scenarios are 5 minutes in duration and tightly standardized by using a scenario template with pre-scripted patient progression. Intervention arm teams will use the aerosol box in all three scenarios while control arm teams will not use the aerosol box. At the end of the entire session, participants will receive an educational debriefing to discuss performance issues, infection control measures, and technical skills using a blended-method approach to debriefing[29]. Doffing will occur in conjunction with a PPE partner, and guided by a standardized PPE doffing checklist to ensure consistency.

Scenario A: *BVM Ventilation* - depicts an adolescent patient with suspected COVID-19, presenting with progressive respiratory distress and desaturation. Participants will be directed to initiate BVM ventilation with a HEPA filter as per guidelines for managing COVID-19 pediatric patients[30]. Aerosolization of particles occurs during manual ventilation, while the mask is on the patient's face. The scenario will last a total of 5 minutes.

Scenario B: ETI - depicts a patient with suspected COVID-19, presenting with progressive respiratory failure requiring intubation. In this scenario, providers will be advised by the team leader not to initiate BVM ventilation, which is consistent with AHA guidelines for adult COVID-19 patients requiring airway management[30]. Participants will be directed to sedate, paralyze and intubate the patient using a videolaryngoscope (e.g. GlideScope™), and provide manual ventilation after intubation with a HEPA filter in place. The scenario will last a maximum of 5 minutes, or until the patient is successfully intubated, whichever is longer.

Scenario C: LMA Insertion - depicts the same patient as in Scenario B. In this scenario, providers will be advised by the team leader to insert an LMA. Participants will be directed to sedate, paralyze and intubate the patient, and provide manual ventilation after LMA insertion with a HEPA filter in place. The scenario will last a maximum of 5 minutes, or until the patient is successfully intubated, whichever is longer.

Data collection will occur immediately after each scenario, prior to and after doffing of PPE (see outcomes below). After data collection, the resuscitation room and airway trainer will be cleaned. Clean PPE will be provided for all participants for each scenario. These measures will ensure there is no incremental accumulation of GloGerm™ particles from prior scenarios. All scenarios will be videotaped from a birds-eye view angle at the foot of the bed.

2.3 Randomization method.

Randomization will occur at the level of the team, stratified by study site and sex of the airway provider (to ensure equal distribution of sex in both arms), and conducted in blocks of 4 to ensure an even distribution of teams across study arms. Randomization packages will be prepared at a central study side using a web-based random number generator. Sequentially numbered recruitment packages provided for each site will contain sealed opaque envelopes (i.e. one envelope per study arm) with study arm assignments and unique identifier codes for participants.

2.4 Protecting against sources of bias.

It will not be possible to blind study participants to study arm allocation due to the nature of the intervention. This should not introduce a significant risk of bias as the outcome measures are highly standardized. To minimize risk of bias, the research associate conducting the photographic analysis (i.e. degree of contamination) will be blinded to study arm allocation.

2.5 Inclusion and exclusion criteria.

Participants from inpatient units, intensive care units, operating room units and emergency departments across all sites will be recruited. Inclusion criteria for the airway provider include: (1) Attending physician or fellow in emergency medicine, intensive care, pediatrics, or anesthesia; and (2) Adult or Pediatric Advanced Life Support certification. Inclusion criteria for the airway assistant include: (1) Attending physician, resident, fellow, nurse or respiratory therapist; and (2) Adult or Pediatric Advanced Life Support certification. Exclusion criteria: (1) Decline to provide informed consent; or (2) Unable to perform tasks required of the role due to physical limitations.

2.6, 2.7 Duration of treatment period and frequency of follow up.

Individual recruitment sessions will be run over the course of the same day. There are no planned follow up sessions.

2.8, 2.9 Outcome measures.

Primary outcome: Surface area of contamination (AOC) quantified from digital photos of GloGerm™ deposited on the airway team members pre and post-doffing. A digital reference grid will be applied over each photo, with mean grey values of areas within each grid "pixel" calculated using Image J (NIH) software[31]. As areas highlighted by GloGerm™ will illuminate more brightly, mean grey value per pixel will be the base unit to quantify degree of contamination relative to the provider's anatomic surface area. This quantitative measure permits comparative analysis between groups or scenarios, and is congruent with accepted measures in the bioimaging informatics[32], digital immunohistochemistry[33], and direct immunofluorescence[34]. Digital images of participants will be captured in a standardized fashion under UV-illumination in a fashion to minimize confounders (brightness, angle, lighting) (see budget). Images will be taken pre- and post-doffing to capture extent of contamination on PPE (pre) and extent of contamination on provider surfaces underneath PPE.

Secondary outcomes: (a) time to successful intubation or LMA insertion (captured by video review by 2 trained and calibrated raters); (b) first pass success rate for ETI and LMA insertion (captured by video review by 2 trained and calibrated raters).

2.10 Sample size.

Sample size estimation is based on primary outcome measure. Given the paucity of quantitative research in this area, we propose a sample of 60 teams (120 participants) in total, or 30 teams (60 participants) per study arm. As each team will receive repeated measures (i.e. 3 scenarios), this sample size allows us to detect a medium effect size (Cohen's d = 0.65), with a significance level of 0.05, a power of 0.8, and a high intra-cluster correlation coefficient (rho = 0.7) to make a conservative estimation. Accounting for missing data due to technical issues, we will recruit 2 extra teams per study group, resulting in a total sample size of 64 teams (128 participants).

2.11 Will health service research issues be addressed?

Our study addresses health service research issues from the point of view of HCP contamination and safety, as well as clinical care of COVID-19 patient using new technology. No additional health service research issues will be addressed.

2.12 Are there likely to be any problems with compliance?

There is a possibility that participants recruited to the intervention arm (i.e. aerosol box) will struggle with performing the airway procedure, which may in turn influence their effort to complete the required task. All participants recruited to the intervention arm will receive comprehensive training, including an opportunity to practice airway procedures with an aerosol box coupled with expert feedback. We will implement a multifaceted approach to ensure compliance with our research protocol

across recruitment sites: (1) development of standard operating procedures and a research protocol guide; (2) submission of recordings of at least one pilot session per site, which will be reviewed by the principal investigator for protocol adherence before recruitment commences; (3) submission of a videotaped recording of one recruitment session per site, three months into the study. Videos will be reviewed by the principal investigator for protocol adherence, with feedback provided to site investigators. We don't anticipate any additional issues with protocol compliance or completion of tasks.

2.13 Loss to follow-up.

This is not applicable as we are not planning any follow-up.

2.45 Recruitment Centers.

Securing space to recruit for simulation-based studies has become more challenging due to institutional restrictions imposed as a result of the COVID-19 pandemic. To overcome this issue and to ensure our study is completed in a timely fashion, the study will be implemented at four INSPIRE network sites (Alberta Children's Hospital, Ste. Justine Hospital, and Children's Hospital of Los Angeles and The Hospital for Sick Children).

2.16, 2.17 Statistical analysis.

Demographic characteristics of participants including sex, profession, years of clinical experience and other identifiers (e.g. height, body surface area) will be reported using descriptive statistics between 2 study arms. For the primary outcome, assuming non-normal distribution of data, we will use Wilcoxon rank sum tests to compare the AOC in 3 different procedures in both airway providers and assistant roles. Analyses to evaluate the degree of contamination pre- and post-doffing of PPE will be conducted separately. Time to procedural completion and first pass success rates will be compared with the Wilcoxon rank sum tests.

2.18 Subgroup analysis.

Although stratifying by sex during randomization will ensure equal distribution of different sex in both groups, the physical stature of each provider may be a factor that influences the extent of contamination. We will conduct secondary analysis to explore if factors other than aerosol box (i.e. sex, body surface area, number of years clinical experience of the airway expert, experience of doing multiple procedures within incubator) will influence the degree of contamination, time to successful intubation and LMA insertion, and first pass success rate for ETI and LMA insertion using mixed-effect linear regression model. The similar secondary analyses focusing on the degree of contamination of the airway assistant will be performed as well while stratified by these factors.

2.19 Has any pilot study been carried out using this design?

Pilot studies conducted by Dr. Matava have established the feasibility of using Glo-Germ™ and the respiratory airway manikin for simulating aerosolization and droplet spray during AGMPs[18, 35]. Dr. Levy's team has developed aerosol box training videos (and training protocols), and pilot tested the use of the aerosol box and simulated cough to aerosolize Glo-Germ. Preliminary data (unpublished) demonstrates minimal contamination of HCPs and the environment with use of the aerosol box (https://www.youtube.com/watch?v=obABuAqFcd4&feature=youtu.be).

References

1. University CfSSaECaJH. COVID-19 Dashboard by the Center for Systems Science and Engineering (CSSE) at Johns Hopkins University 2020 [Available from: https://gisanddata.maps.arcgis.com/apps/opsdashboard/index.html#/bda7594740fd40299423467b48e9ecf6.
2. Guan WJ, Ni ZY, Hu Y, Liang WH, Ou CQ, He JX, et al. Clinical Characteristics of Coronavirus Disease 2019 in China. N Engl J Med. 2020;382(18):1708-20.
3. Livingston E, Bucher K. Coronavirus Disease 2019 (COVID-19) in Italy. JAMA. 2020.
4. Meng L, Qiu H, Wan L, Ai Y, Xue Z, Guo Q, et al. Intubation and Ventilation amid the COVID-19 Outbreak: Wuhan's Experience. Anesthesiology. 2020.
5. Wang D, Hu B, Hu C, Zhu F, Liu X, Zhang J, et al. Clinical Characteristics of 138 Hospitalized Patients With 2019 Novel Coronavirus-Infected Pneumonia in Wuhan, China. JAMA. 2020.
6. Grasselli G, Zangrillo A, Zanella A, Antonelli M, Cabrini L, Castelli A, et al. Baseline Characteristics and Outcomes of 1591 Patients Infected With SARS-CoV-2 Admitted to ICUs of the Lombardy Region, Italy. JAMA. 2020.
7. Wu Z, McGoogan JM. Characteristics of and Important Lessons From the Coronavirus Disease 2019 (COVID-19) Outbreak in China: Summary of a Report of 72314 Cases From the Chinese Center for Disease Control and Prevention. JAMA. 2020.
8. Huang C, Wang Y, Li X, Ren L, Zhao J, Hu Y, et al. Clinical features of patients infected with 2019 novel coronavirus in Wuhan, China. Lancet. 2020;395(10223):497-506.
9. Lockhart SL, Naidu JJ, Badh CS, Duggan LV. Simulation as a tool for assessing and evolving your current personal protective equipment: lessons learned during the coronavirus disease (COVID-19) pandemic. Can J Anaesth. 2020.
10. Tran K, Cimon K, Severn M, Pessoa-Silva CL, Conly J. Aerosol generating procedures and risk of transmission of acute respiratory infections to healthcare workers: a systematic review. PLoS One. 2012;7(4):e35797.
11. Lai HY. Aerosol Box: Protecdts Healthcare Providers During Endotracheal Intubation 2020 [Available from: https://sites.google.com/view/aerosolbox/home?authuser=1.
12. Canelli R, Connor CW, Gonzalez M, Nozari A, Ortega R. Barrier Enclosure during Endotracheal Intubation. N Engl J Med. 2020.
13. Leyva Moraga FA, Leyva Moraga E, Leyva Moraga F, Juanz Gonzalez A, Ibarra Celaya JM, Ocejo Gallegos JA, et al. Aerosol box, An Operating Room Security Measure in COVID-19 Pandemic. World J Surg. 2020.
14. Ibrahim M, Khan E, Babazade R, Simon M, Vadhera R. Comparison of the Effectiveness of Different Barrier Enclosure Techniques in Protection of Healthcare Workers During Tracheal Intubation and Extubation. A A Pract. 2020;14(8):e01252.
15. Dalli J, Khan MF, Marsh B, Nolan K, Cahill RA. Evaluating intubation boxes for airway management. Br J Anaesth. 2020.
16. Gore RK, Saldana C, Wright DW, Klein AM. Intubation Containment System for Improved Protection From Aerosolized Particles During Airway Management. IEEE J Transl Eng Health Med. 2020;8:1600103.
17. Alix-Seguin L, Levy A. Guide d'utilisation du Splash Guard à l'urgence CHU Sainte-Justine chez un patient avec un diagnostic suspecté ou confirmé d'infection à l'urgence du CHU Sainte-Justine au virus SARS-CoV-2 (COVID-19) 2020 [Available from: http://www.urgencehsj.ca/wp-content/uploads/Guide-dutilisation-du-Splash-Guard-urgence-HSJ-COVID-19-2020.04.24.pdf.
18. Matava CT, Yu J, Denning S. Clear plastic drapes may be effective at limiting aerosolization and droplet spray during extubation: implications for COVID-19. Can J Anaesth. 2020.

19. Jazuli F, Bilic M, Hanel E, Ha M, Hassall K, Trotter BG. Endotracheal intubation with barrier protection. Emerg Med J. 2020.
20. Simpson JP, Wong DN, Verco L, Carter R, Dzidowski M, Chan PY. Measurement of airborne particle exposure during simulated tracheal intubation using various proposed aerosol containment devices during the COVID-19 pandemic. Anaesthesia. 2020.
21. Begley JL, Lavery KE, Nickson CP, Brewster DJ. The aerosol box for intubation in coronavirus disease 2019 patients: an in-situ simulation crossover study. Anaesthesia. 2020.
22. Cheng A, Auerbach M, Hunt EA, Chang TP, Pusic M, Nadkarni V, et al. Designing and conducting simulation-based research. Pediatrics. 2014;133(6):1091-101.
23. Cheng A, Kessler D, Mackinnon R, Chang TP, Nadkarni VM, Hunt EA, et al. Reporting guidelines for health care simulation research: Extensions to the CONSORT and STROBE statements. BMJ Simulation and Technology Enhanced Learning. 2016:bmjstel-2016-000124.
24. Judson SD, Munster VJ. Nosocomial Transmission of Emerging Viruses via Aerosol-Generating Medical Procedures. Viruses. 2019;11(10).
25. Fowler RA, Guest CB, Lapinsky SE, Sibbald WJ, Louie M, Tang P, et al. Transmission of severe acute respiratory syndrome during intubation and mechanical ventilation. American journal of respiratory and critical care medicine. 2004;169(11):1198-202.
26. Christian MD, Loutfy M, McDonald LC, Martinez KF, Ofner M, Wong T, et al. Possible SARS coronavirus transmission during cardiopulmonary resuscitation. Emerg Infect Dis. 2004;10(2):287-93.
27. Organization WH. Modes of transmission of virus causing COVID-19: implications for IPC precaution recommendations 2020 [Available from: https://www.who.int/news-room/commentaries/detail/modes-of-transmission-of-virus-causing-covid-19-implications-for-ipc-precaution-recommendations.
28. Duggan L. Associate Professor, University of Ottawa.
29. Eppich W, Cheng A. Promoting Excellence and Reflective Learning in Simulation (PEARLS): development and rationale for a blended approach to health care simulation debriefing. Simul Healthc. 2015;10(2):106-15.
30. Edelson DP, Sasson M, Chan PS, Atkins DL, Aziz K, Becker LB, et al. Interim Guidance for Basic and Advanced Life Support in Adults, Childrens, and Neonates With Suspected or Confirmed COVID-19: From the Emergency Cardiovascular Care Committee and the Get With the Guildelines-Resuscitation Adult and Pediatric Task Forces of the American Heart Association in Collaboration with the American Academy of Pediatrics, American Association for Respiratory Care, American College of Emergency Physicians, The Society of Critical Care Anesthesiologists, and American Society of Anesthesiiologist: Supporting Organizations: American Association of Critical Care Nurses and National EMS Physicians. Circulation. 2020.
31. Schindelin J, Rueden CT, Hiner MC, Eliceiri KW. The ImageJ ecosystem: An open platform for biomedical image analysis. Mol Reprod Dev. 2015;82(7-8):518-29.
32. Eliceiri KW, Berthold MR, Goldberg IG, Ibanez L, Manjunath BS, Martone ME, et al. Biological imaging software tools. Nat Methods. 2012;9(7):697-710.
33. Laurinaviciene A, Plancoulaine B, Baltrusaityte I, Meskauskas R, Besusparis J, Lesciute-Krilaviciene D, et al. Digital immunohistochemistry platform for the staining variation monitoring based on integration of image and statistical analyses with laboratory information system. Diagn Pathol. 2014;9 Suppl 1:S10.
34. Chessel A. An Overview of data science uses in bioimage informatics. Methods. 2017;115:110-8.
35. Matava C, Collard V, Siegel J, Denning S, Li T, Du B, et al. Use of a high-flow extractor to reduce aerosol exposure in tracheal intubation. British Journal of Anaesthesia. 2020;125(4):e363-e6.
