# Supplementary material for: Aerosol Box Use in Reducing Health Care Worker Contamination During Airway Procedures (AIRWAY Study): A Simulation-Based Randomized Clinical Trial
Source: JAMA Netw Open. 2023 Apr 12;6(4):e237894. doi: 10.1001/jamanetworkopen.2023.7894 (PMC10099073; doi:10.1001/jamanetworkopen.2023.7894)
Supplement: Supplement 3. — Nonauthor Collaborators [file jamanetwopen-e237894-s003.pdf]

Supplemental Online Content: Nonauthor Collaborators
*First name, last name, and suffix (if applicable) are required and will appear in PubMed.

***Group Name(s): International Network for Simulation-based Pediatric Innovation, Research and Education (INSPIRE) Aerosol-Generating Medical Procedure (AGMP) Investigators**							
***First Name and Middle Initial(s)**	***Last Name**	***Suffix (eg, Jr, III)**	Academic Degrees	Institution	Location (city, state/province, country)	Role or Contribution, eg, chair, principal investigator	Group (if more than 1 Group listed in the byline) and/or Subgroup (eg, Steering Committee)
Donovan	Duncan		MD, FRCPC	University of Calgary / Alberta Children's Hospital	Calgary, Alberta, Canada	Investigator (data collection)	INSPIRE AGMP Investigators
Jabeen	Fayyaz		MD	University of Toronto / The Hospital for Sick Children	Toronto,Ontario,Canada	Investigator (data collection)	INSPIRE AGMP Investigators
Mireille	Garhib		MD, FRCPC	University of Toronto / The Hospital for Sick Children	Toronto,Ontario,Canada	Investigator (data collection)	INSPIRE AGMP Investigators
Theophilus	Tackey		BSc, BA	University of Toronto/York University	Toronto,Ontario,Canada	Investigator (data collection)	INSPIRE AGMP Investigators
