# Supplementary material for: Aerosol Box Use in Reducing Health Care Worker Contamination During Airway Procedures (AIRWAY Study): A Simulation-Based Randomized Clinical Trial
Source: JAMA Netw Open. 2023 Apr 12;6(4):e237894. doi: 10.1001/jamanetworkopen.2023.7894 (PMC10099073; doi:10.1001/jamanetworkopen.2023.7894)
Supplement: Supplement 4. — Data Sharing Statement [file jamanetwopen-e237894-s004.pdf]

Data Sharing Statement

Cheng. Aerosol Box Use in Reducing Health Care Worker Contamination During Airway Procedures (AIRWAY Study). *JAMA Netw Open*. Published April 12, 2023. doi:10.1001/jamanetworkopen.2023.7894

Data
Data available: No

Additional Information
Explanation for why data not available: Data includes photos of participants which we would like to keep confidential
